# A Skeletal Muscle Model of Infantile-onset Pompe Disease with Patient-specific iPS Cells

**DOI:** 10.1038/s41598-017-14063-y

**Published:** 2017-10-18

**Authors:** Takeshi Yoshida, Tomonari Awaya, Tatsuya Jonouchi, Ryo Kimura, Shigemi Kimura, Takumi Era, Toshio Heike, Hidetoshi Sakurai

**Affiliations:** 1 Center for iPS Cell Research and Application (CiRA), Kyoto University, Kyoto, 606-8507 Japan; 2 Department of Pediatrics, Kyoto University Graduate School of Medicine, Kyoto, 606-8507 Japan; 3 Department of Anatomy and Developmental Biology, Kyoto University Graduate School of Medicine, Kyoto, 606-8501 Japan; 4 Kumamoto City Child Development Support Center, Kumamoto, 862-0971 Japan; 5 Department of Cell Modulation, Institute of Molecular Embryology and Genetics (IMEG), Kumamoto University, Kumamoto, 860-8556 Japan

## Abstract

Pompe disease is caused by an inborn defect of lysosomal acid α-glucosidase (GAA) and is characterized by lysosomal glycogen accumulation primarily in the skeletal muscle and heart. Patients with the severe type of the disease, infantile-onset Pompe disease (IOPD), show generalized muscle weakness and heart failure in early infancy. They cannot survive over two years. Enzyme replacement therapy with recombinant human GAA (rhGAA) improves the survival rate, but its effect on skeletal muscle is insufficient compared to other organs. Moreover, the patho-mechanism of skeletal muscle damage in IOPD is still unclear. Here we generated induced pluripotent stem cells (iPSCs) from patients with IOPD and differentiated them into myocytes. Differentiated myocytes showed lysosomal glycogen accumulation, which was dose-dependently rescued by rhGAA. We further demonstrated that mammalian/mechanistic target of rapamycin complex 1 (mTORC1) activity was impaired in IOPD iPSC-derived myocytes. Comprehensive metabolomic and transcriptomic analyses suggested the disturbance of mTORC1-related signaling, including deteriorated energy status and suppressed mitochondrial oxidative function. In summary, we successfully established an *in vitro* skeletal muscle model of IOPD using patient-specific iPSCs. Disturbed mTORC1 signaling may contribute to the pathogenesis of skeletal muscle damage in IOPD, and may be a potential therapeutic target for Pompe disease.

## Introduction

Pompe disease (OMIM 232300, glycogen storage disease type II or acid maltase deficiency) is one of the lysosomal storage disorders, caused by an inborn defect of lysosomal acid α-glucosidase (GAA). GAA is the only enzyme that can degrade glycogen into glucose in the lysosomes. Thus, the lack of GAA causes abnormal accumulation of glycogen within the lysosomes, primarily in the skeletal muscle and heart^1^. Patients with Pompe disease show an extremely wide spectrum in the severity of their symptoms depending on the residual amount of GAA activity, and are generally classified into two categories according to time of onset^2^, infantile-onset Pompe disease (IOPD) and late-onset (LOPD). Patients with IOPD develop generalized muscle weakness and heart failure in early infancy, and almost all the patients cannot survive over two years^3,4^. On the other hand, patients with LOPD, having partial defects of GAA, slowly develop progressive skeletal muscle weakness, often resulting in ventilator dependence and shortened lifespans^5^. The only treatment currently available is enzyme replacement therapy (ERT) with recombinant human GAA (rhGAA), which dramatically improves the survival rate in patients with IOPD^6,7^. However, the limitations of ERT have become increasingly evident. ERT is very effective on cardiac symptoms, but its effect on skeletal muscle symptoms is limited, and many patients eventually become dependent on artificial ventilation. In addition, emerging anti-rhGAA antibodies that attenuate therapeutic response to ERT is another serious problem for lifelong treatment^8,9^. Thus, the development of a novel therapeutic approach or adjunctive therapy to the current ERT is urgently needed.

The pathogenesis of skeletal muscle damage in Pompe disease has not been fully elucidated. Formerly, lysosomal rupture due to glycogen accumulation and release of its lytic enzymes into the cytoplasm were considered as the explanation of muscle damage^10,11^. Recent studies of GAA knockout mice or muscle biopsies from patients with LOPD demonstrated that secondary autophagic dysfunction plays an important role in progressive muscle damage^12–15^. However, such autophagic dysfunction is not remarkable in the muscle of patients with IOPD despite the extremely enlarged lysosomes^16^, suggesting the possibility of a different patho-mechanism of muscle damage in LOPD or GAA knockout mice.

Human induced pluripotent stem cells (iPSCs) are very powerful tools for disease modeling because of their differentiation potential into various types of tissue^17^. In Pompe disease, several disease models using patient iPSCs were recently reported^18–22^. However, an iPSC-based skeletal muscle model of IOPD has not been established. To address some of the unsolved clinical problems described above, an efficient skeletal muscle model of IOPD is particularly needed. In this study, we generated iPSCs from three patients with IOPD and differentiated them into myocytes. As a result, differentiated myocytes showed the expansion of glycogen-filled lysosomes, the pathological hallmark of Pompe disease, which was dose-dependently restored by rhGAA treatment. Furthermore, we demonstrated that mammalian/mechanistic target of rapamycin complex 1 (mTORC1) signaling and energy metabolism were affected by lysosomal glycogen accumulation in our model. This means that our iPS-based skeletal muscle model partly clarified the patho-mechanism of skeletal muscle damage in IOPD.

## Results

### Generation of MyoD-transfected iPSC from healthy controls and patients with IOPD

**Figure 1:**
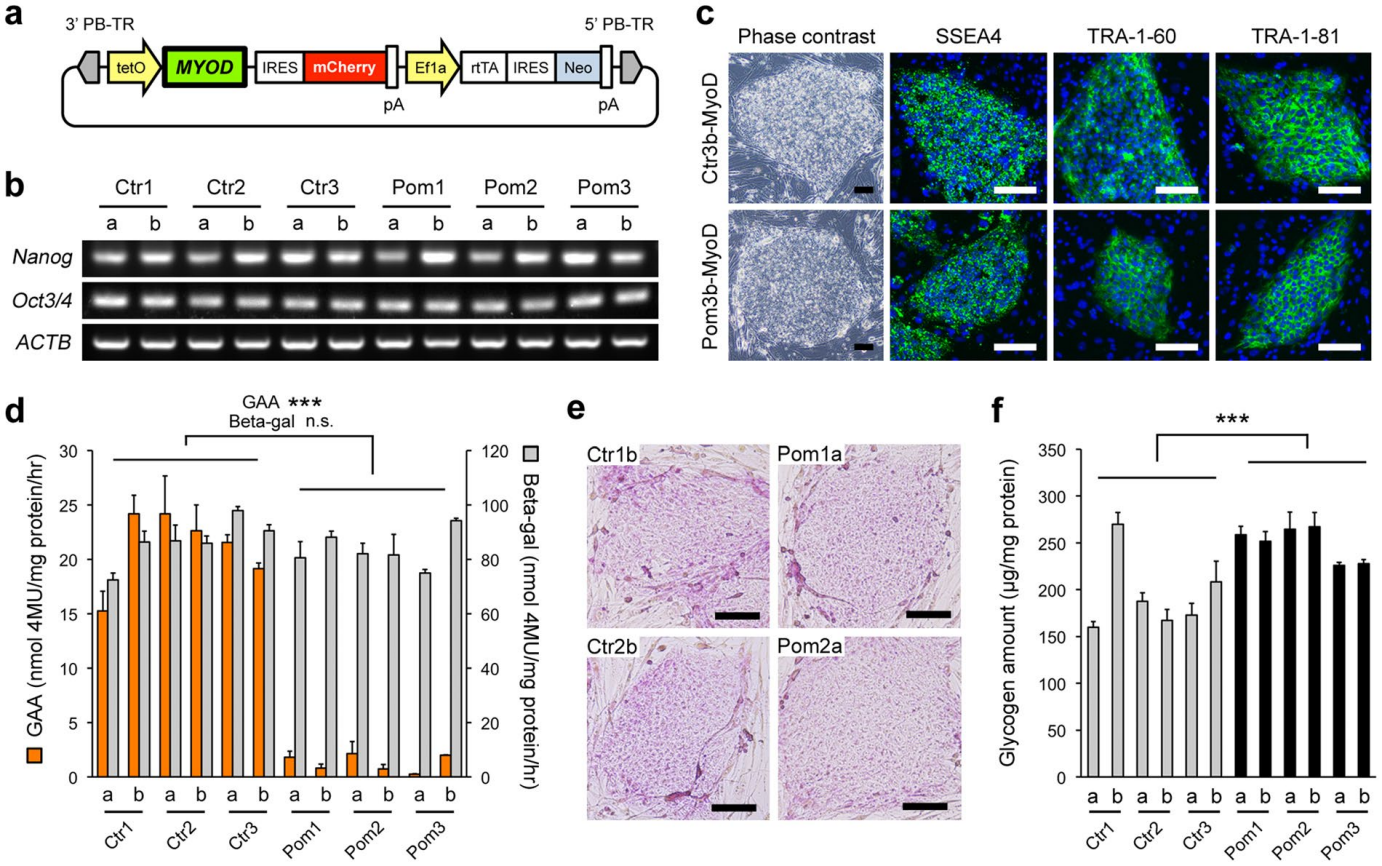
Generation and characterization of MyoD-transfected iPSCs (iPSCs^MyoD^) from healthy controls and patients with infantile-onset Pompe disease. (**a**) Construction of the piggyBac vector for tetracycline-inducible *MyoD* expression. Abbreviations: PB-TR, PiggyBac terminal repeat; IRES, internal ribosome entry site; Ef1a, elongation factor 1 alpha promoter; rtTA, reverse tetracycline transactivator; Neo, neomycin resistant gene; pA, poly A. (**b**) RT-PCR analysis of pluripotency markers in iPSC^MyoD^ derived from Ctr1-3 and Pom1-3. (**c**) Phase contrast microscopic images (left row) and IF for pluripotency markers (SSEA4, TRA-1-60, and TRA-1-81; green) (right 3 rows) in representative undifferentiated Ctr and Pom iPSC^MyoD^ lines. Nuclei were stained with DAPI (blue). Scale bar = 100 µm. (**d**) Lysosomal enzymatic activities in undifferentiated iPSC^MyoD^ (n = 3 experiments). Orange bars and left y-axis represent the activities of GAA. Grey bars and right y-axis represent the activities of acid beta-galactosidase (Beta-gal) as an internal control. (**e**) Bright field microscopic images of PAS stain in representative undifferentiated Ctr and Pom iPSC^MyoD^. Scale bar = 100 µm. (**f**) Quantitative analysis of glycogen amounts adjusted for protein in undifferentiated iPSC^MyoD^ (n = 3 experiments).

We generated iPSCs from three healthy controls, designated as “Ctr1-3”, and three unrelated patients with IOPD, “Pom1-3”, using previously described methods^23–25^. The karyotypes of all iPSC lines were confirmed as normal (Supplementary Fig. S1). We had previously established an efficient myogenic differentiation system from human iPSCs using the piggyBac vector for tetracycline-inducible expression of *MyoD*, which is a master regulator of myogenic differentiation^26^. Using the technique previously described, we introduced tetracycline-inducible *MyoD* into all six iPSC lines (designated as “iPSC^MyoD^”) (Fig. 1a). The induction efficiency of MyoD was calculated by flow cytometric analysis of mCherry expression. The efficiency of all lines was higher than 80% and was not different between Ctr and Pom iPSC lines (Supplementary Fig. S2a,b).

Next, we selected two clones (“a” and “b”) with high myogenic differentiation potential from each iPSC^MyoD^ line. These clones from both Ctr and Pom iPSC^MyoD^ expressed pluripotency markers. The expression of *Nanog* and *Oct3/4* was confirmed by reverse transcription-polymerase chain reaction (RT-PCR) analysis (Fig. 1b), while that of stage specific embryonic antigen-4 (SSEA4), tumor-related antigen (TRA)-1-60, and TRA-1-81 was confirmed by immunofluorescence (IF) (Fig. 1c). In addition, these cells presented a small round shape and high nuclear to cytoplasm ratios, resembling the morphology of the original iPSCs (Fig. 1c). These data indicated that these *MyoD*-transfected clones retained pluripotent characteristics.

### A partial phenotype of Pompe disease in undifferentiated Pom iPSC^MyoD^

First, we analyzed the lysosomal enzymatic activities in iPSCs^MyoD^. The activity of GAA, the causative enzyme of Pompe disease, was much lower in Pom iPSC^MyoD^ than in Ctr (Fig. 1d). In contrast, the activity of beta galactosidase, another lysosomal enzyme we used as an internal control, was similar in both groups (Fig. 1d). To evaluate whether undifferentiated Pom iPSC^MyoD^ presented Pompe disease-specific phenotypes, we analyzed the glycogen state by Periodic Acid-Schiff (PAS) stain, which can detect polysaccharides, including glycogen, and by directly measuring glycogen amounts. PAS stain was slightly positive in the cytoplasm of cells from both groups, showing no remarkable difference (Fig. 1e). Glycogen amount of cell lysate adjusted for the protein amount was slightly higher in Pom iPSC^MyoD^ (Fig. 1f), suggesting that small amount of glycogen accumulated in undifferentiated Pom iPSC^MyoD^, which was undetectable by microscopic inspection.

### Efficient myogenic differentiation of iPSC^MyoD^

**Figure 2:**
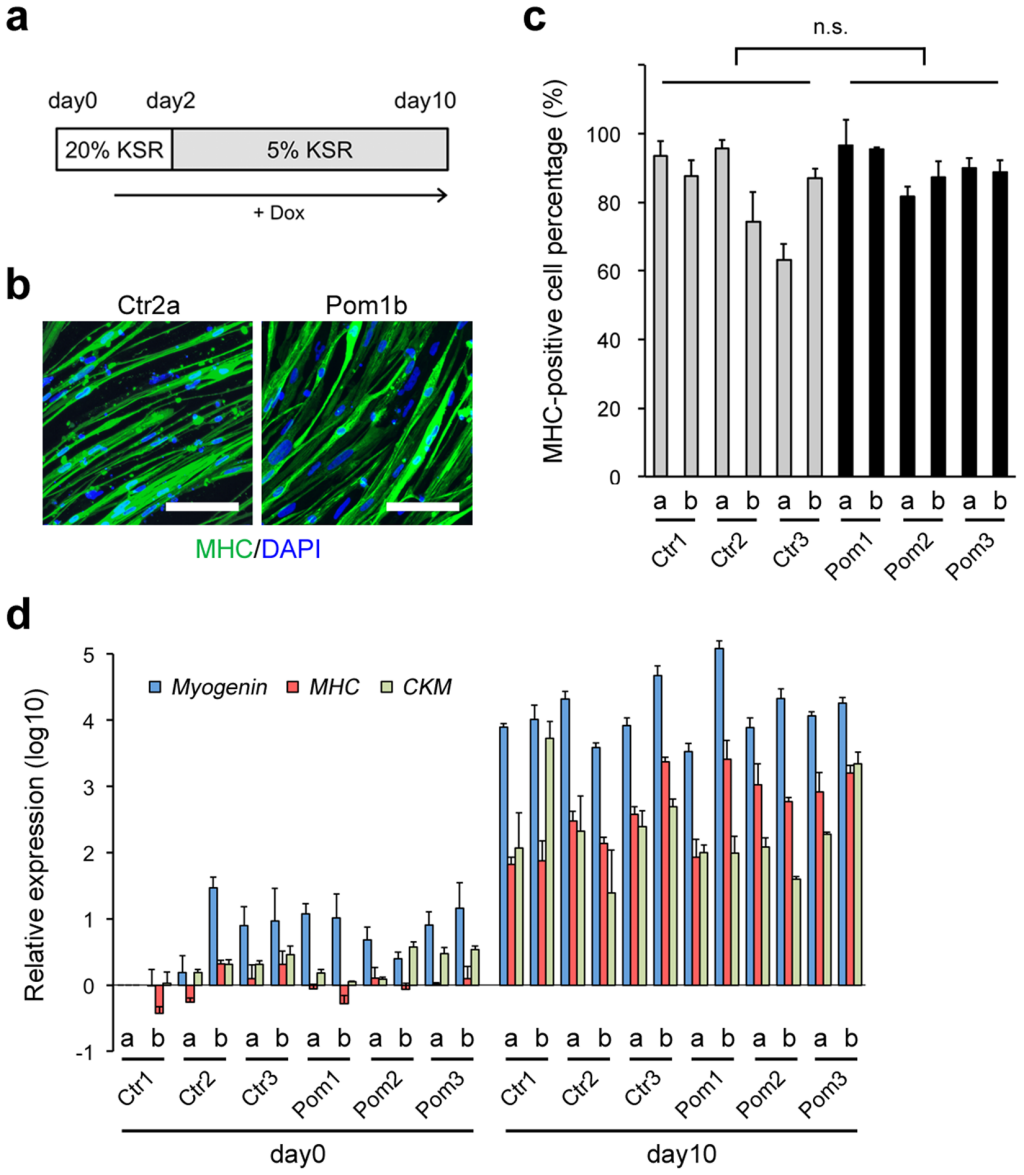
Characterization of differentiated myocytes derived from iPSC^MyoD^. (**a**) A scheme of myogenic differentiation of iPSC^MyoD^ with Dox-inducible *MyoD* overexpression. Cells were cultured with human iPSC medium containing 20% knockout serum replacement (KSR) for the first 2 days. Dox was started on day 1. (**b**) IF for MHC (green) in representative Ctr and Pom iPSC^MyoD^-derived myocytes. Nuclei were stained with DAPI. Scale bar = 100 µm. (**c**) Percentage of MHC-positive cells per total cells at day 10 of myogenic differentiation in all cell lines (n = 3 microscopic fields). (**d**) Quantitative RT-PCR analysis for myogenic markers (*Myogenin*, blue; *MHC*, red; and *CKM*, green) at day 0 (undifferentiated iPSC^MyoD^) and day 10 of myogenic differentiation in all cell lines. The graph logarithmically represents relative gene expression compared to the level of Ctr1a iPSC^MyoD^ at day 0 (n = 3 experiments). Ubiquitin C was used as an internal control.

To investigate the phenotype of Pompe disease in iPSC-derived myocytes, we performed myogenic differentiation of iPSC^MyoD^ through doxycycline (Dox)-inducible MyoD overexpression (Fig. 2a). On day 10, differentiated cells from both Ctr and Pom iPSC^MyoD^ were spindle-shaped and mostly positive for myosin heavy chain (MHC), a marker of mature myocytes, in IF (Fig. 2b). The differentiation efficiency, calculated as the ratio of MHC-positive cells to total cells, ranged from 65% to 95%, showing no statistical difference between Ctr and Pom iPSC groups (Fig. 2c). We also analyzed the expression of three myogenic differentiation markers, *myogenin*, *MHC*, and *creatine kinase M-type* (*CKM*), using quantitative RT-PCR. Compared to undifferentiated iPSC^MyoD^ (day 0), all lines of differentiated cells (day 10) showed much higher expression levels of myogenic markers (Fig. 2d). These data demonstrated that our myogenic differentiation strategy via *MyoD* overexpression enables us to obtain comparable skeletal muscle cells (myocytes) from Pom iPSC^MyoD^ as well as Ctr iPSC^MyoD^.

### Lysosomal glycogen accumulation in Pom iPSC^MyoD^-derived myocytes

**Figure 3:**
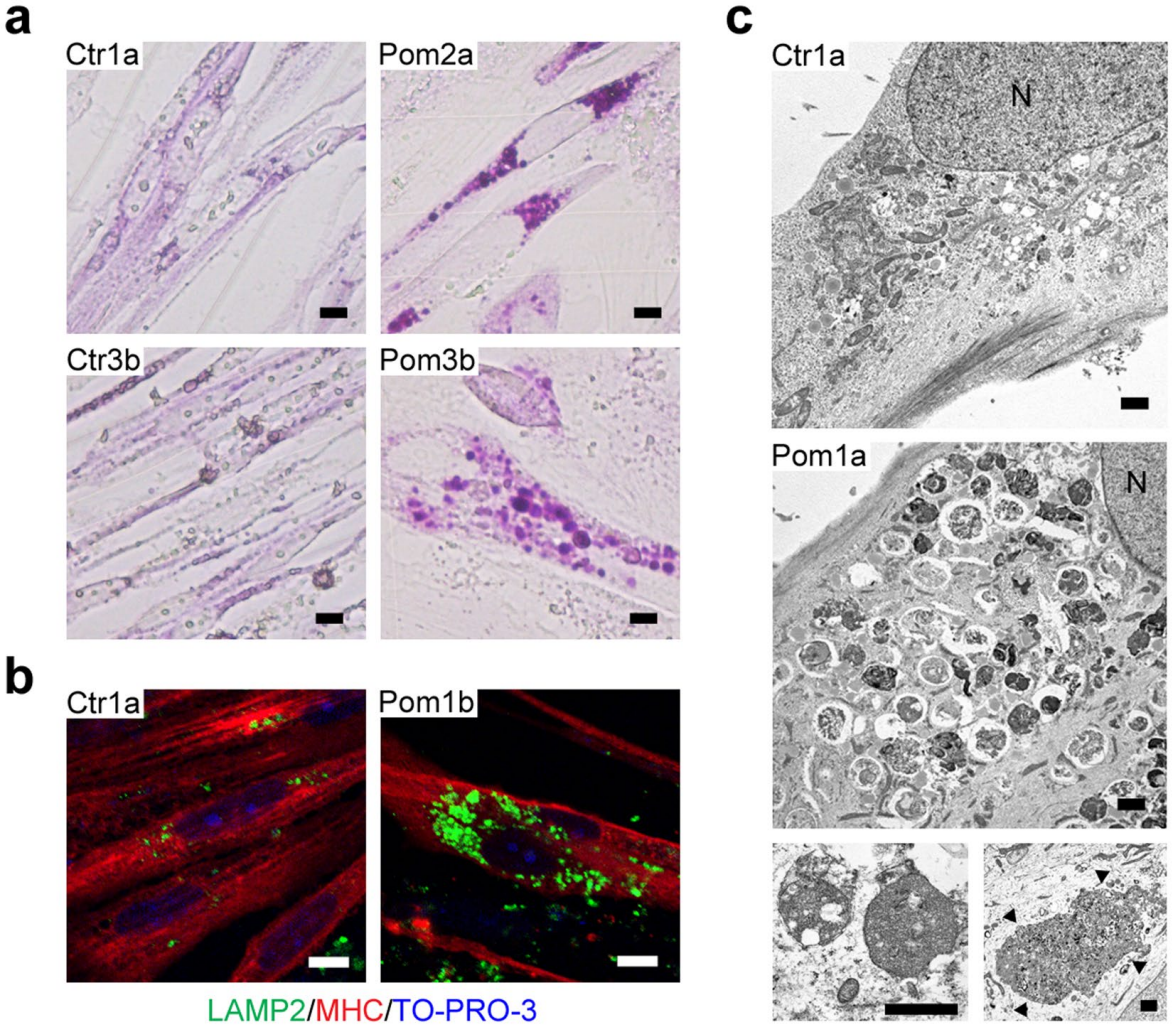
Lysosomal glycogen accumulation in Pom iPSC^MyoD^-derived myocytes. (**a**) Bright field microscopic images of PAS stain in representative Ctr and Pom iPSC^MyoD^-derived myocytes. Scale bar = 10 µm. (**b**) Confocal microscopic images of IF for LAMP2 (green) and MHC (red) in representative Ctr and Pom iPSC^MyoD^-derived myocytes. Nuclei were stained with TO-PRO-3. Scale bar = 10 µm. (**c**) Electron microscopic images in representative Ctr and Pom iPSC^MyoD^-derived myocytes. The most lower panels are more and less magnified images. Some Pom1a iPSC^MyoD^-derived myocytes contain an extremely large glycogen-filled lysosome (arrowheads). A capital letter “N” represents a nucleus. Scale bar = 1 µm.

Glycogen analysis, IF, and electron microscopic observation were performed to analyze glycogen accumulation in iPSC^MyoD^-derived myocytes. PAS stain showed that the entire cytoplasm was weekly stained in a uniform manner in Ctr iPSC^MyoD^-derived myocytes. In contrast, many strongly stained granules, ranging up to 8 µm in diameter, were located around the nuclei in Pom iPSC^MyoD^-derived myocytes (Fig. 3a). These PAS-positive granules observed in Pom iPSC^MyoD^-derived myocytes were also positive for lysosome-associated membrane protein 2 (LAMP2; lysosomal marker) in IF (Fig. 3b). Electron microscopy analysis revealed numerous round membrane-bound structures packed with small dots around the nuclei in Pom iPSC^MyoD^-derived myocytes (Fig. 3c). These abnormal structures were indicative of enlarged lysosomes packed with glycogen. Intriguingly, some Pom iPSC^MyoD^-derived myocytes contained extraordinarily large lysosomes (Fig. 3c, arrowheads). From these data, we concluded that glycogen had abnormally accumulated in the lysosomes in Pom iPSC^MyoD^-derived myocytes, which is the pathognomonic feature of Pompe disease.

### Analysis of lysosomal glycogen accumulation with transient glucose deprivation in Pom iPSC^MyoD^-derived myocytes

**Figure 4:**
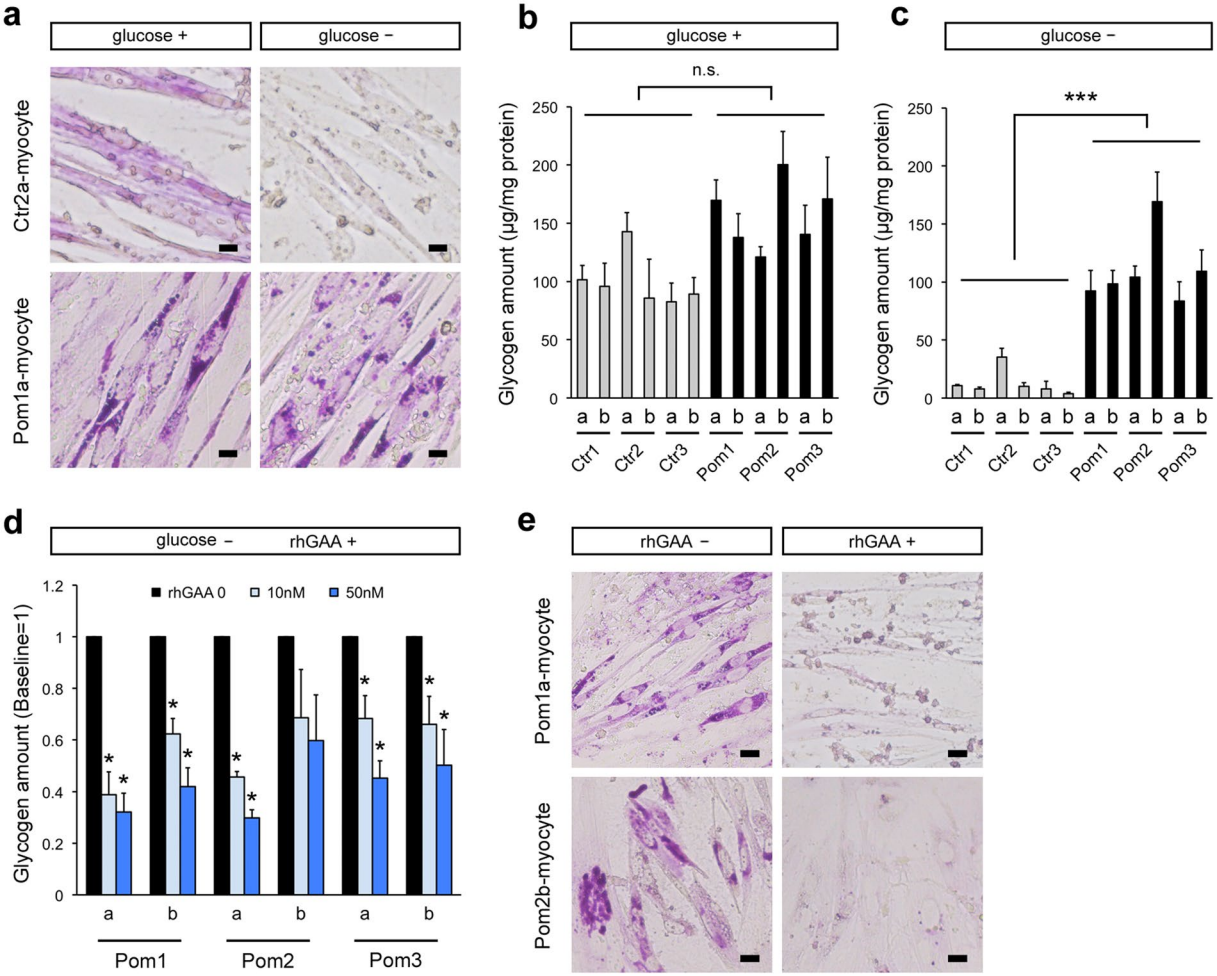
Analysis of lysosomal glycogen accumulation with transient glucose deprivation in Pom iPSC^MyoD^-derived myocytes. (**a**) Representative bright field microscopic images of PAS stain in Ctr and Pom iPSC^MyoD^-derived myocytes. The left row (designated as “glucose+”) shows myocytes cultured in normal glucose-containing medium. The right row (“glucose–”) shows myocytes after 24 h culture with glucose-free medium. Scale bar = 10 µm. (**b**) Quantitative analysis of glycogen amounts adjusted for protein amounts in iPSCMyoD-derived myocytes in normal glucose-containing medium (n = 3 experiments). (**c**) Quantitative analysis of glycogen amounts adjusted for protein amounts in iPSC^MyoD^-derived myocytes after 24 h of culture with glucose-free medium (n = 3 experiments). (**d**) Quantitative analysis of glycogen amounts adjusted for protein after 24 h of glucose deprivation and 3 days of treatment with rhGAA (0, 10, and 50 nM) in Pom iPSC^MyoD^-derived myocytes. The graph represents the relative ratio to the baseline glycogen amount (no rhGAA). Asterisks indicate a significant difference compared to the baseline (n = 3 experiments). (**e**) Representative bright field microscopic images of PAS stain in Pom iPSC^MyoD^-derived myocytes after 24 h of glucose deprivation without rhGAA treatment (left row) and with rhGAA treatment (right row). Scale bar = 20 µm.

Normally, glycogen is mainly located in the cytoplasm and only a small amount of glycogen is detected in the lysosomes^27,28^. To analyze lysosomal glycogen alone, culture medium was replaced with glucose-free medium 24 h prior to the glycogen analysis so that cytoplasmic glycogen would be consumed. As expected, Ctr iPSC^MyoD^-derived myocytes showed almost no PAS staining after glucose deprivation (Fig. 4a). In contrast, enlarged lysosomes packed with glycogen in Pom iPSC^MyoD^-derived myocytes remained strongly positive after glucose deprivation due to the lack of GAA (Fig. 4a). Quantitative analysis of glycogen had similar results with the PAS stain: without transient glucose deprivation, glycogen amounts were not different between Pom and Ctr iPSC^MyoD^-derived myocytes due to the existence of cytoplasmic glycogen(Fig. 4b); meanwhile, after glucose deprivation, remaining glycogen amounts were significantly higher in Pom myocytes than control (Fig. 4c). Furthermore, to evaluate the effect of rhGAA in this model, rhGAA was added for the last 3 days of myogenic differentiation at three different concentrations, 0, 10, and 50 nM. After glucose deprivation, accumulated lysosomal glycogen was significantly improved in a dose-dependent manner (Fig. 4d) except for Pom2b line, where glycogen accumulation was particularly prominent (Fig. 4b,c). PAS stain after glucose deprivation showed complete improvement of lysosomal glycogen accumulation after 3 days of rhGAA treatment (Fig. 4e). Without glucose deprivation, rhGAA treatment ameliorated strongly-stained granules observed in PAS stain (Supplementary Fig. S3a). Together, these results demonstrate that our skeletal muscle model successfully recapitulates the pathophysiological features of Pompe disease, and it can be used to assess the efficacy of drug treatment (summarized in Supplementary Fig. S3b–g).

### mTORC1 activation is suppressed and energy metabolism is altered in Pom iPSC^MyoD^-derived myocytes

**Figure 5:**
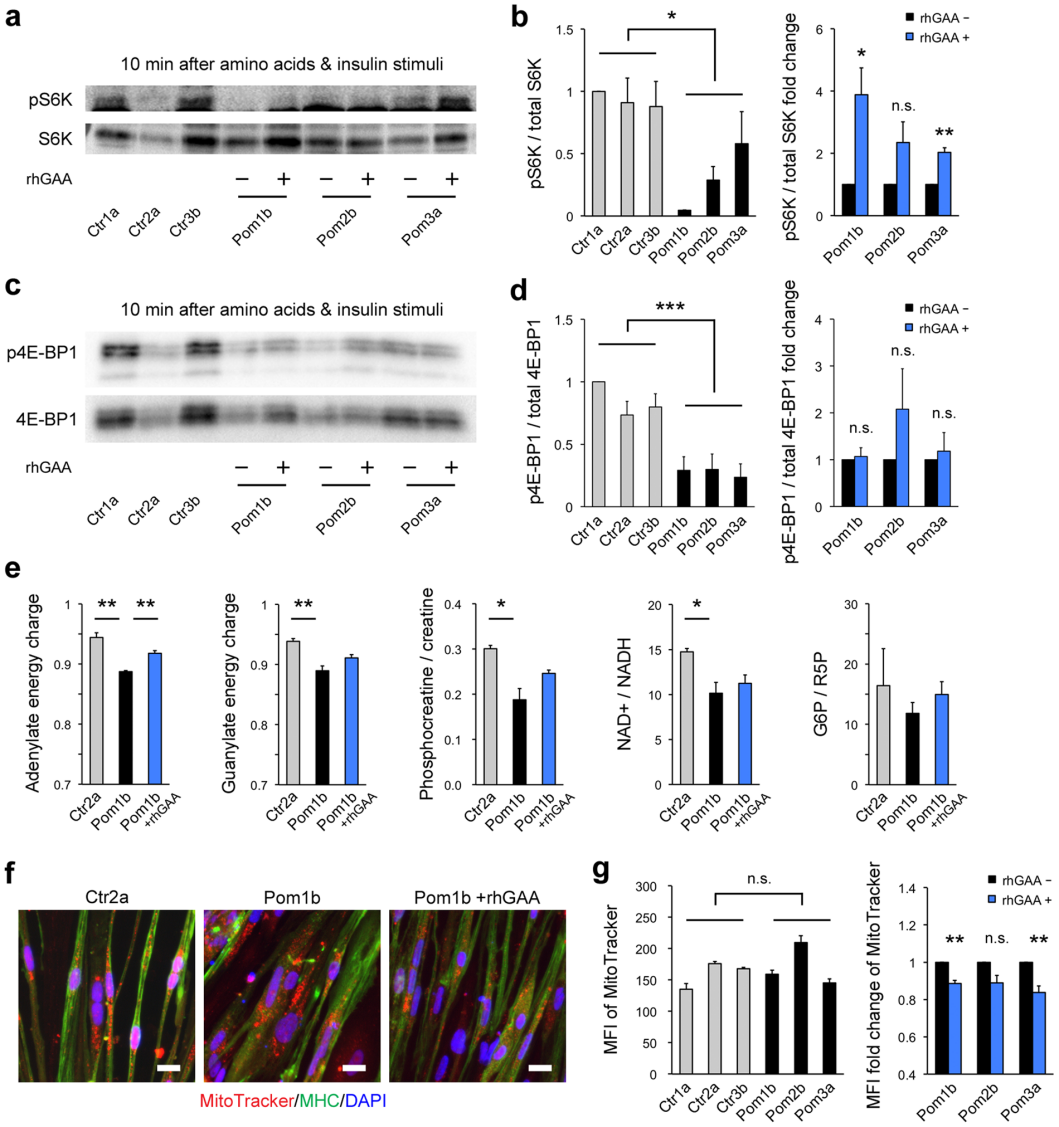
Suppressed mTORC1 activation and deterioration of energy metabolism in Pom iPSC^MyoD^-derived myocytes. (**a**) Representative blots of Western blot analysis for pS6K and total S6K after 10 min exposure to amino acid and insulin in protein extracts from iPSC^MyoD^-derived myocytes. Pom iPSC^MyoD^-derived myocytes were treated with or without rhGAA. ACTB was used as a loading control. (**b**) Quantitative densitometric analysis of western blots (n = 3 experiments). The left graph shows pS6K/total S6K ratio normalized to Ctr1a in iPSC^MyoD^-derived myocytes. The right graph shows fold change of pS6K/total S6K ratio with rhGAA rescue. (**c**) Representative blots of Western blot analysis for p4E-BP1 and total 4E-BP1 after 10 min exposure to amino acid and insulin in protein extracts from iPSC^MyoD^-derived myocytes. Pom iPSC^MyoD^-derived myocytes were treated with or without rhGAA. (**d**) Quantitative densitometric analysis of western blots (n = 3 experiments). The left graph shows p4E-BP1/total 4E-BP1 ratio normalized to Ctr1a in iPSC^MyoD^-derived myocytes. The right graph shows fold change of p4E-BP1/total 4E-BP1 ratio with rhGAA rescue. (**e**) Representative metabolic indices from metabolomic analysis in iPSC^MyoD^-derived myocytes (n = 3 experiments). Abbreviations: NAD+, nicotinamide adenine dinucleotide+; NADH, NAD hydride; G6P, glucose 6-phosphate; R5P, ribose 5-phosphate. (**f**) Representative microscopic images of MitoTracker Red (red) and IF for MHC (green) in Ctr and Pom iPSC^MyoD^-derived myocytes. Nuclei were stained with DAPI. Scale bar = 20 µm. (**g**) The left graph shows mean fluorescent intensity (MFI) of MitoTracker Red (integrated MitoTracker fluorescent signals adjusted for MHC-positive area) in iPSC^MyoD^-derived myocytes (n = 3 microscopic fields). The right graph shows fold change of MFI with rhGAA rescue in Pom iPSC^MyoD^-derived myocytes.

**Figure 6:**
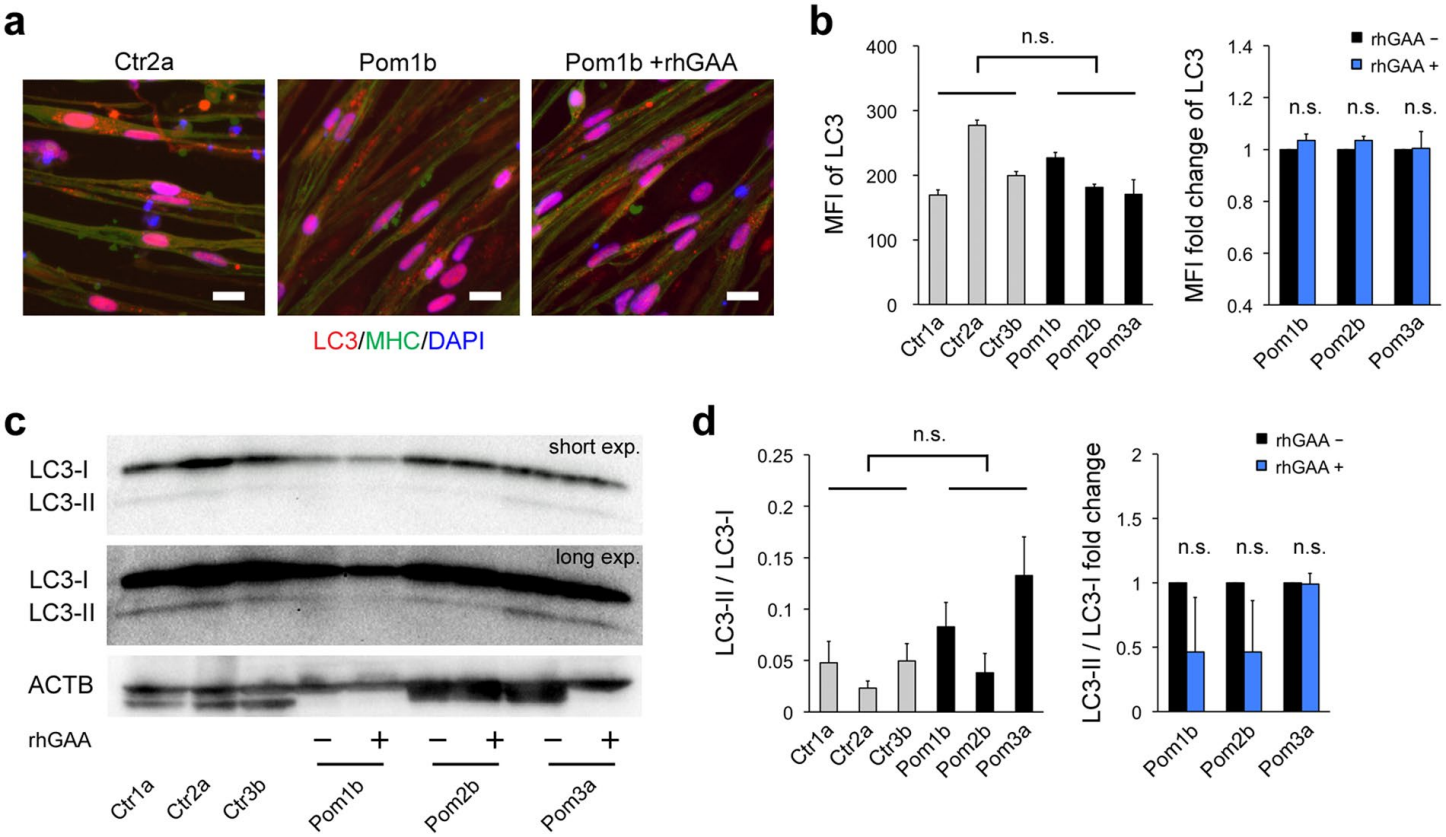
No significant autophagic accumulation in Pom iPSC^MyoD^-derived myocytes. (**a**) Representative microscopic images of immunofluorescence for LC3 (red; autophagic marker) and MHC (green) in iPSC^MyoD^-derived myocytes. Pom iPSC^MyoD^-derived myocytes were cultured without or with 1 µM rhGAA for 3 days. Nuclei were stained with DAPI. Scale bar = 20 µm. (**b**) MFI of LC3 (integrated LC3 fluorescent signals adjusted for MHC-positive area) in iPSC^MyoD^-derived myocytes (left graph) and the fold change of MFI with rhGAA rescue (right) (n = 3 microscopic fields). (**c**) Representative blots of Western blot analysis for LC3 in protein extracts from iPSC^MyoD^-derived myocytes. Abbreviation: exp., exposure. (**d**) Quantitative densitometric analysis of western blots (n = 3 experiments). The left graph shows LC3-II/LC3-I ratio in iPSC^MyoD^-derived myocytes. The right graph shows fold change of LC3-II/LC3-I ratio with rhGAA rescue.

An increasing number of studies recently revealed that lysosomes are not just waste disposals, but also participates in signaling pathways. mTORC1, a key regulator of the cellular metabolic network, is activated on the lysosomal surface^29^ and plays a central role in lysosomal signaling^30^. To investigate the influence of lysosomal glycogen accumulation on mTORC1 activity in our model, we selected one clone with better myogenic differentiation from each iPSC line and performed western blot analysis for phosphorylation of 2 targets of mTORC1, p70 ribosomal S6 kinase 1 (S6K) and eukaryotic initiation factor 4E-binding protein 1 (4E-BP1), induced by the exposure to amino acids and insulin, the best-characterized readout of mTORC1 activation^31^. Western blot analysis showed that both proteins were significantly less phosphorylated in Pom iPSC^MyoD^-derived myocytes than that in control (Fig. 5a–d) (baseline phosphorylation levels of both proteins were showed in Supplementary Fig. S4). To eliminate clonal variation of iPSC lines, we evaluated the response to rhGAA individually. Reduced phosphorylation was partially rescued by the addition of rhGAA in S6K (Fig. 5b), but not in 4E-BP1 (Fig. 5d) (phosphorylated bands were confirmed by pretreatment of rapamycin, mTORC1 inhibitor, in Fig. S5). These results indicate that lysosomal glycogen accumulation is associated with suppressed mTORC1 activation. Due to the great diversity of intracellular metabolic activities associated with mTORC1, we selected two clones that show the best myogenic differentiation efficiency from each Ctr and Pom group, and performed a comprehensive metabolomic analysis (Supplementary Table S3). It revealed that Pom iPSC^MyoD^-derived myocytes showed decreased levels of adenylate energy charge, defined as (ATP + ADP/2)/(ATP + ADP + AMP) ratio, guanylate energy charge, (GTP + GDP/2)/(GTP + GDP + GMP) ratio, and phosphocreatine/creatine ratio (a marker of cytosolic energy state) (Fig. 5e). Adenylate energy charge was significantly improved by the addition of rhGAA in Pom myocytes (Fig. 5e). These results suggest that Pom myocytes showed deteriorated cellular energy metabolism. As for other metabolic indices, NAD+/NADH ratio (a marker of mitochondrial oxidative function) was decreased in Pom iPSC^MyoD^-derived myocytes; while G6P/R5P (glucose 6-phosphate/ribose 5-phosphate) ratio (a marker of glycolysis and pentose-phosphate pathway) was similar in both groups (Fig. 5e), suggesting that deteriorated energy metabolism in Pom myocytes is mainly due to the mitochondrial dysfunction. Furthermore, we performed MitoTracker staining to assess the condition of mitochondria in iPSC^MyoD^-derived myocytes (Fig. 5f). Calculated mean fluorescence intensity (MFI) of MitoTracker signals were not different between Pom and Ctr myocytes. However, when we compared each Pom clone with or without rhGAA to avoid clonal variation, the addition of rhGAA significantly decreased the MFI of MitoTracker in two of the three Pom lines (Fig. 5g). These data suggest lysosomal glycogen accumulation has some influence on the mitochondrial condition in Pom iPSC^MyoD^-derived myocytes. Meanwhile, MFI of microtubule-associated protein 1 light chain 3 (LC3), a marker of autophagy, in IF of Pom iPSC^MyoD^-derived myocytes did not change with the addition of rhGAA (Fig. 6a,b). Similarly, the ratios of LC3-II to LC3-I, a marker of autophagy induction, were evaluated by Western blot analysis, showing no difference between Ctr and Pom groups, or between without and with rhGAA treatment in each Pom clone (Fig. 6c,d). These data suggested that aberrant autophagic accumulation did not occur in Pom myocytes, as observed in the muscle specimens of patients with IOPD^16^. Taken together, these results suggest that suppressed mTORC1 activation is associated with the deterioration of energy metabolism in Pom iPSC^MyoD^-derived myocytes, which can be partly attributed to the mitochondrial dysfunction.

### rhGAA rescue alters gene expression profile in Pom iPSC^MyoD^-derived myocytes

**Figure 7:**
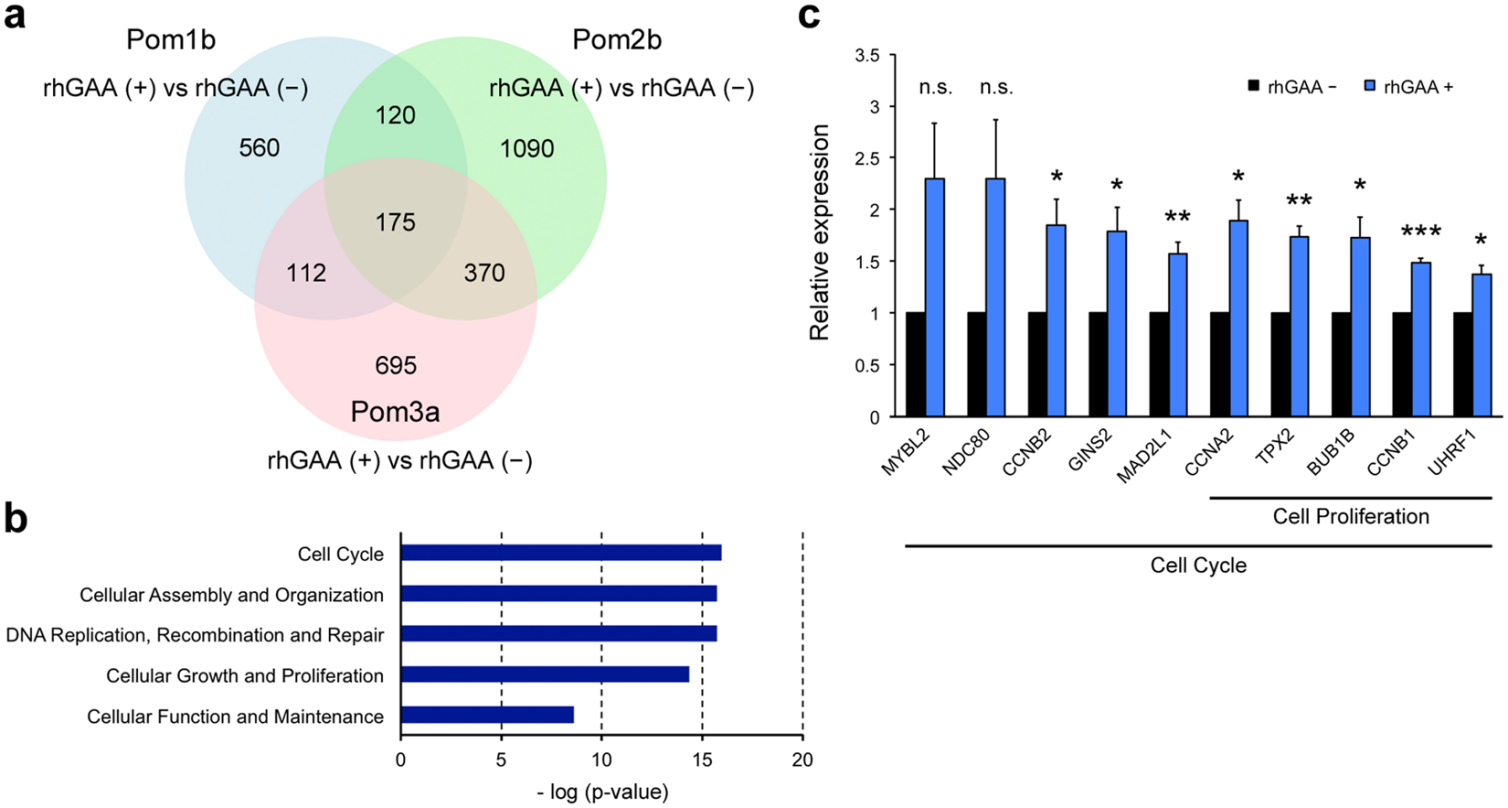
Transcriptomic analysis in Pom iPSC^MyoD^-derived myocytes. (**a**) A Venn diagram of genes identified as differentially expressed between Pom iPSC^MyoD^-derived myocytes treated with and without rhGAA. Numbers in the diagram represent gene numbers. (**b**) The top 5 networks associated with common 175 genes identified from the Venn diagram according to the pathway analysis. (**c**) Quantitative RT-PCR analysis for genes related to cell cycle or cell proliferation (*MYBL2*, MYB proto-oncogene like 2; *NDC80*, NDC80, kinetochore complex component; *CCNB2*, cyclin B2; *GINS2*, GINS complex subunit 2; *MAD2L1*, mitotic arrest deficient 2 like 1; *CCNA2*, cyclin A2; *TPX2*, TPX2, microtubule nucleation factor; *BUB1B*, BUB1 mitotic checkpoint serine/threonine kinase B; *CCNB1*, cyclin B1; *UHRF1*, ubiquitin like with PHD and ring finger domains 1). The graph represents relative gene expression compared to the level of Pom iPSC^MyoD^-derived myocytes without rhGAA treatment (n = 3 cell lines). Beta-actin was used as an internal control.

To further elucidate the influence of suppressed mTORC1 activation, we performed transcriptomic analysis in Pom iPSC^MyoD^-derived myocytes. To eliminate clonal variation of iPSC lines, we compared each pair of Pom myocytes with or without rhGAA and extracted common genes among the three datasets (Fig. 7a). As a result, 175 genes were identified and categorized into the following networks of molecular and cellular function using pathway analysis: Cell cycle; cellular assembly and organization; DNA replication, recombination, and repair; cellular growth and proliferation; and cellular function and maintenance (The top 5 are listed in Fig. 7b). Most of these identified networks were associated with mTORC1 downstream signaling^32,33^. We randomly selected some genes related to cell cycle or cell proliferation among the common 175 genes, and confirmed the expression by quantitative RT-PCR. As a result, expressions of 8 of 10 genes we confirmed were significantly increased with rhGAA treatment, and those of 2 genes showed tendency to be increased with rhGAA treatment (p-values were 0.071 and 0.087 in *MYBL2* and *NDC80* respectively) (Fig. 7c). These results support that mTORC1 signaling was altered in Pom iPSC^MyoD^-derived myocytes due to aberrant accumulation of lysosomal glycogen.

## Discussion

When researching human diseases, high quality disease models are essential for further understanding of the disease mechanism and developing novel treatment approaches. In Pompe disease, several *in vivo* and *in vitro* models have been developed so far. Among them, GAA-knockout (KO) mice are the most widely used and provided us with invaluable insights into the patho-mechanism of this disease. The homozygous GAA-KO mouse certainly shows glycogen accumulation in the lysosomes of the skeletal muscle and heart soon after birth, but they can grow up to adult age and the clinical symptoms are relatively mild, unlike the severe human type, IOPD^34,35^. From a pathological viewpoint, GAA-KO mouse shows characteristic accumulation of autophagic debris subsequent to lysosomal dysfunction in the affected muscle fibers^12,14^, which rather resembles LOPD than IOPD^16^. *In vitro* muscle models established from GAA-KO mouse^36,37^ or GAA-KO mouse-iPSCs^38^ have a similar limitation of discrepancy between species. On the other hand, *in vitro* human muscle models established by isolating primary myoblasts from patients’ biopsy specimens are desirable disease models for tailor-maid research. However, muscle biopsies are invasive, particularly for infantile patients, and have become unnecessary for diagnosis or evaluation of treatment response in the majority of patients with Pompe disease because of technological advances^39^. Thus, we need to develop a novel and efficient skeletal muscle model to investigate the patho-mechanism in IOPD.

After the establishment of human iPSCs^23^, disease models using patient-specific iPSCs have been increasingly reported^17^. iPSCs can be generated from the patients’ own somatic cells, including fibroblasts or peripheral blood cells, with minimal invasion and can be differentiated to various types of tissue^25^. Hence, they can provide important insights into the patho-mechanism in affected tissues, which are usually difficult to obtain. Moreover, due to the unlimited proliferation potential of iPSCs, iPSC-based disease models can be a platform for high-throughput screening of drugs or small molecules^40^. In this study, we used MyoD overexpression strategy to establish a skeletal muscle model from patient’s iPSCs. This strategy enabled us to obtain structurally and physiologically comparable multi-nucleated myocytes in a relatively short period^26^, and it can be applied to modeling of various skeletal muscle diseases including Duchenne muscular dystrophy^41,42^, Miyoshi myopathy^26^, and carnitine palmitoyltransferase II deficiency^43^. Given that myogenic differentiation without gene transduction from iPSCs is time-consuming and not highly efficient^44,45^; the MyoD-overexpression strategy is a reasonable option for modeling muscle diseases. In this study, we generated iPSCs from patients with IOPD and differentiated them into myocytes using this strategy. The myocytes from patients’ iPSCs showed lysosomal glycogen accumulation, the pathognomonic feature of Pompe disease. We further demonstrated that such lysosomal glycogen accumulation improved in a dose-dependent manner after rhGAA treatment. To our knowledge, this is the first report describing a skeletal muscle model of IOPD using patient-specific iPSCs, where treatment response can be quantitatively assessed.

Lysosomes are membrane-bound organelles, containing various hydrolytic enzymes, and were long considered only responsible for degrading and recycling cellular wastes. However, it became increasingly evident that lysosomes have much wider functions. Recent evidence showed that lysosomes regulated various intracellular signals according to the cellular conditions^29^. A multi-component kinase, mTORC1, a master regulator of the cellular metabolism and growth, plays a central role in such lysosomal signaling via phosphorylation of several downstream targets^30,46^. We here analyzed phosphorylation of 2 targets of mTORC1, S6K and 4E-BP1, by exposure to amino acids and insulin, and demonstrated that mTORC1 activation was significantly reduced in myocytes derived from Pom iPSC^MyoD^. This is consistent with recent reports of suppressed mTORC1 activation in fibroblasts from patients with IOPD^47^, GAA-knockdown C2C12 myoblasts, or GAA-KO mice^48,49^. In our study, responses to rhGAA treatment were different between S6K and 4E-BP1, which may be attributed to the more complicated regulation of phosphorylation of 4E-BP1 than that of S6K^50^. Although it has been thought that accumulated glycogen impairs lysosomal function by elevating lysosomal pH^29,36^, further investigation is still necessary to clarify the precise mechanism of mTORC1 inactivation in IOPD.

Although cell-type specific roles of mTORC1 is not well understood, mTORC1 is indispensable for a number of bio-physiological processes in the skeletal muscle such as cell growth, mitochondrial oxidative functions, and autophagy^51–53^. Mice with muscle-specific inactivation of mTORC1 develop severe muscle phenotype and die at the early age^54–56^. These conditional KO mice showed metabolic changes in skeletal muscles, including impaired oxidative metabolism, altered mitochondrial regulation, and glycogen accumulation. According to the metabolomic analysis in our model, Pompe disease myocytes showed deteriorated energy status and suppressed oxidative metabolism. Moreover, mitochondrial signals altered with rhGAA treatment. These data suggests that disturbed mTORC1 activation has some influence on the mitochondrial regulation in Pompe myocytes. Mice with muscle-specific ablation of raptor, an essential component of mTORC1, showed swollen mitochondria in the damaged muscles^54^. Studies of other lysosomal storage diseases also indicated the accumulation of morphologically aberrant mitochondria^57,58^. Collectively, impaired mTORC1 activation and mitochondrial dysfunction can play some roles in the pathogenesis of skeletal muscle phenotype in IOPD. Notably, such impaired mTORC1 signaling was somewhat reversible by rhGAA treatment in our model. Thus, early initiation of enzyme replacement would be beneficial, as indicated from a clinical study^59^.

With respect to the gene expression profiling in our muscle model, pathways associated with cell cycle or cell proliferation were altered by rhGAA treatment. These are some of the major downstream pathways of mTORC1^32,33^ and might affect the *in vivo* proliferation of myoblasts in patients with Pompe disease. However, MyoD-overexpression strongly inhibits cell proliferation. Thus our model is not suitable to analyze these pathways. Since the association between mTORC1 signaling and the patho-mechanism of Pompe disease has not been fully elucidated, further investigation will be necessary.

In conclusion, we successfully established an *in vitro* skeletal muscle model of IOPD using patient-specific iPSCs, which can be used to quantitatively evaluate the response of rhGAA. Furthermore, we showed suppressed mTORC1 activation and altered downstream signaling in IOPD using our muscle model. Disturbed mTORC1 signaling can contribute to the pathogenesis of the skeletal muscle damage in IOPD and can be a potential therapeutic target for not only Pompe disease, but also whole lysosomal storage disorders.

## Methods

### Ethical approval

All experimental protocols in the study were approved by the Ethics Committee Graduate School and Faculty of Medicine Kyoto University (approval number #R0091 and #G259). The study was performed conforming to the guidelines of the Declaration of Helsinki and conducted after obtaining written informed consents.

### Cell lines and cell culture

All human iPSC lines used in this study were generated from fibroblasts. Pom2 iPSC line and Ctr1 iPSC line were kindly provided by Dr Shigemi Kimura, Dr Takumi Era (Kumamoto University), and Dr. Shinya Yamanaka (Kyoto University), respectively. The other iPSC lines were established as previously described. Three patients with IOPD were clinically diagnosed by the almost complete lack of GAA activity. Upon examination of the sequence of all exons and exon-intron junctions, Pom1 patient only presented the single mutation of c.1880C > T in *GAA*. Pom2 patient presented the *GAA* mutations, c.796 C > T and c.1316 T > A. Pom3 patient presented with c. 1798C > T and c.2481 + 1 G > A. All iPSC lines were cultured on mouse feeder cells in Primate ES Cell Medium (Reprocell, Yokohama, Japan) containing 10 ng/mL of recombinant human basic fibroblast growth factor (bFGF) (Oriental Yeast, Tokyo, Japan).

### *MyoD* transfection

We constructed the piggyBac (PB)-based vector for tetracycline-inducible expression of *MyoD*, and inserted the vector into iPSCs, as previously described^41^. This vector also expressed mCherry in a tetracycline-inducible manner; and the neomycin resistance gene constitutively (Fig. 1a).

### Protein isolation and analysis of lysosomal enzymatic activities

Cultured cells were trypsinized and washed twice with phosphate buffered saline (PBS). Then, cell pellets were sonicated on ice three times and lysed in radio-immunoprecipitation assay (RIPA) buffer (Nacalai Tesque, Kyoto, Japan) containing 1% (v/v) Protease Inhibitor Cocktail (PIC) (Nacalai Tesque). Protein concentrations of the lysates were measured using Pierce BCA Protein Assay Kit (Thermo Fisher Scientific, Waltham, MA, USA) following the manufacturer’s instructions. Prior to the analysis of lysosomal enzymatic activities, stock solutions of 4-methylumbelliferyl (MU)-α-D-glucopyranoside (synthetic GAA substrate) (Sigma-Aldrich, St. Louis, MO, USA) and 4-MU-β-D-galactopyranoside (synthetic β-D-galactosidase substrate) (Nacalai Tesque) were prepared at 70 mM in dimethyl sulfoxide (Sigma-Aldrich). The lysates equivalent to 30 µg of protein were incubated at 37 °C for 1 h together with 1.5 mM 4-MU-α-D-glucopyranoside or 1.5 mM 4-MU-β-D-galactopyranoside in citrate-phosphate buffer (pH 3.7) in a total 100 µL reaction mixture. Reactions were stopped with 200 µL ethylenediaminetetraacetic acid buffer (pH 11.4) and fluorescence levels were measured with EnVision^®^ Multilabel Plate Reader (PerkinElmer, Waltham, MA, USA) (excitation wavelength, 360 nm; emission, 450 nm). A series of different concentrations of 4-methylumbelliferone (Nacalai Tesque) were prepared in every experiment for standardization. As repeated freeze and thaw cycles of the lysates considerably reduced enzymatic activities (data not shown), both protein isolation and enzymatic analysis were performed within the same day.

### PAS stain

PAS stain was performed with the PAS Staining Kit (Muto Pure Chemicals, Tokyo, Japan) following the manufacturer’s instructions. Briefly, cells were fixed with 10.5% (w/v) formaldehyde and treated with 1% (w/v) periodic acid for 10 min at room temperature. After the cells were washed three times with distilled water, they were treated with Schiff’s reagent for 30 min at 37 °C. Staining reaction was stopped by three treatments with sulfurous acid solution. The samples were completely dried and observed with a DP73 microscope (Olympus) in bright field.

### Glycogen analysis

Cultured cells were trypsinized and washed twice with PBS. Then, cell pellets were sonicated on ice in distilled water. Protein concentrations of the lysates were determined using the Pierce BCA Protein Assay Kit (Thermo Fisher Scientific). The lysates were diluted to cell type-specific protein concentrations so that the glycogen amount would be within the detection range of the Glycogen Assay Kit (BioVision, Milpitas, CA, USA). Then, glycogen amounts were analyzed following the manufacturer’s instructions. Fluorescence levels were measured with EnVision Multilabel Plate Reader (PerkinElmer).

### *In vitro* myogenic differentiation and rhGAA rescue experiment

For myogenic differentiation, we modified a previously reported protocol (Fig. 2a). Briefly, iPSC^MyoD^ were trypsinized, dissociated to single cells, and seeded on Matrigel (BD Biosciences, San Diego, CA, USA)-coated plates at a density from 4 × 10^3^ to 5 × 10^4^ cells/cm^2^ (cell line-specific) in 20% (v/v) knockout serum replacement (KSR) human iPSC medium with 10 µM Y-27632. After 24 h, Dox (LKT Laboratories, St. Paul, MN, USA) was added at 1 µg/mL. On day 2, the medium was replaced with high glucose (4.5 g/L) Dulbecco’s modified Eagle’s medium (DMEM) (Nacalai Tesque) containing 5% (v/v) KSR (Thermo Fisher Scientific), 1 µg/mL Dox, 1 mM L-glutamine (Thermo Fisher Scientific), and 0.1 mM 2-mercaptoethanol (2-ME) (Thermo Fisher Scientific). The medium was changed daily. For the transient glucose deprivation experiment, the medium was replaced with glucose-free DMEM/Ham’s F-12 (Nacalai Tesque) containing 1 µg/mL Dox, 1 mM L-glutamine and 0.1 mM 2-ME for 24 h prior to the glycogen analysis. For the rhGAA rescue experiment, 1 µM Myozyme (rhGAA) (Sanofi Genzyme, Cambridge, MA, USA) was added to the medium for the last 3 days unless otherwise specified.

### RNA isolation and RT-PCR

Total RNA was isolated using ReliaPrep RNA Cell Miniprep System (Promega, Madison, WI, USA) according to the manufacturer’s instructions. Isolated RNA was treated with DNase and then reverse transcribed using RevaTra Ace kit (Toyobo, Osaka, Japan). Quantitative PCR for myogenic markers was performed on a StepOnePlus™ instrument (Thermo Fisher Scientific) with SYBR Green dye (Thermo Fisher Scientific). Quantitative PCR for confirmation of the transcriptomic analysis was performed using the TaqMan Gene Expression Assays (Thermo Fisher Scientific). Assays were performed in duplicate with the ABI 7900HT Fast Real-Time PCR System according to the manufacturer’s protocol (Thermo Fisher Scientific). Relative gene expression was calculated by the comparative Ct method using β-actin as an endogenous control. PCR primers are listed in Supplementary Table S1.

### Immunofluorescence of cultured cells

Cells were fixed with PBS containing with 2% (w/v) paraformaldehyde for 10 min at 4 °C. Fixed samples were blocked with Blocking One (Nacalai Tesque) for 45 min at 4 °C and incubated overnight at 4 °C with primary antibodies diluted in 10% (v/v) Blocking One in PBS-T (PBS with 0.2% (v/v) Triton X-100 solution (Nacalai Tesque)). The samples were then washed 3 times with PBS-T and incubated for 1 h at room temperature with secondary antibodies diluted in 10% (v/v) Blocking One in PBS-T. Mitochondria were stained with MitoTracker Red CMXRos (Thermo Fisher Scientific) according to the manufacturer’s instructions. Nuclei were stained with DAPI (1:5000; Sigma-Aldrich) or TO-PRO-3 (1:1000; Thermo Fisher Scientific). The samples were observed with BZ-X700 (KEYENCE, Osaka, Japan) or LSM710NLO confocal microscope (Carl Zeiss, Oberkochen, Germany). The MFI of MitoTracker Red or LC3 in MHC-positive area was calculated using BZ-X Analyzer software (KEYENCE). Antibodies are listed in Supplementary Table S2.

### Electron microscopy

Samples were chemically fixed and observed by Tokai Electron Microscopy, Inc. (Nagoya, Japan).

### mTORC1 activation assay

Myocytes were cultured in D-MEM with Sodium Pyruvate, without Amino Acids (WAKO, Osaka, Japan) for 1 h to lower mTORC1 activity. The cells were then cultured with DMEM containing amino acids and 100 nM insulin (Nacalai Tesque) for 10 min. After washing with cold PBS, the cells were lysed in RIPA buffer containing 1% (v/v) PIC and thoroughly sonicated on ice. Proteins were isolated from the lysate as described above. The isolated proteins (20 µg) were separated by electrophoresis on NuPAGE Novex 3–8% Tris-Acetate Protein Gel (Thermo Fisher Scientific) at 150 V for 60 min for S6K and pS6K, or on Extra PAGE One Precast Gel 15% (Nacalai Tesque) at 300 V for 30 min for 4E-BP1, p4E-BP1 and LC3, and transferred to a nitrocellulose membrane using an iBlot system (Thermo Fisher Scientific) with the program, P0, 9 min. The membrane was blocked with PBS-T containing 1% (w/w) skim milk and then incubated with primary antibody solution at 4 °C overnight. After washing with PBS-T three times, the membrane was incubated with secondary antibody solution for 1 h at room temperature. The blots were developed by Pierce Western Blotting Substrate Plus (Thermo Fisher Scientific) or ImmunoStar LD (WAKO). The bands were digitally detected by ChemiDoc XRS+ (Bio-Rad, Hercules, CA, USA) and quantified by Quantity One software (Bio-Rad).

### Rapamycin analysis

Myocytes were cultured in amino acid-free DMEM for 1 h with 200 nM Rapamycin (WAKO). The cells were then cultured with DMEM containing amino acids and 100 nM insulin for 10 min. Protein isolation and western blotting of pS6K and p4E-BP1 were performed as described above.

### Metabolomic analysis

Metabolomic analysis of myocytes derived from iPSCs^MyoD^ was performed using capillary electrophoresis time-of-flight mass spectrometry by Human Metabolome Technologies Inc. (Tsuruoka, Japan) as previously reported^60^.

### Gene expression profiling and microarray data analysis

Microarray analysis was performed by using the Agilent SurePrint G3 Human GE v2 8 × 60 K Microarray (G4851B) following the manufacturer’s standard protocols (Agilent Technologies, Tokyo, Japan). Total RNA (100 ng) from samples was labeled with Cy3 using Low Input Quick Amp RNA Labeling kit, One-Color (Agilent Technologies). Cy3-labeled cRNA (600 ng) was fragmented and hybridized at 65 °C for 17 hours. Then, the slides were scanned on the Agilent Microarray scanner (G2565CA) and data were extracted with Agilent Feature Extraction software version 11.0.1.1 (Agilent Technologies). Microarray data were analyzed using R and Limma package from Bioconductor (http://www.r-project.org). Expression values were background corrected using the normexp method, and between-arrays normalization was performed using the quantile method. All Agilent control probes and low expressed probes were removed. Agilent probe IDs were annotated using the BioMart (Ensembl version 84). Differential expressed genes were detected with threshold *P* < 0.01 and fold change >1.2. The pathway analysis was performed to identify enriched molecular and cellular functions using Ingenuity Pathway Analysis (IPA, QIAGEN Redwood City, CA, USA; www.qiagen.com/ingenuity).

### Statistical analysis

Statistical analyses were performed using Scheffé’s multiple comparison method when comparing Pompe disease *vs*. control groups. To analyze the response to different doses of rhGAA, Williams’ multiple comparison test was used. To compare averages from two samples, Student’s t-test was used. Data was shown as mean ± standard error. *p < 0.05, **p < 0.01, ***p < 0.001.

### Data availability

The datasets generated and/or analyzed during the current study are available from the corresponding author on reasonable request.

## Electronic supplementary material

Supplementary information
