# Supplementary material for: A Skeletal Muscle Model of Infantile-onset Pompe Disease with Patient-specific iPS Cells
Source: Sci Rep. 2017 Oct 18;7:13473. doi: 10.1038/s41598-017-14063-y (PMC5647434; doi:10.1038/s41598-017-14063-y)
Supplement: Supplementary file 1 — Supplementary information [file 41598_2017_14063_MOESM1_ESM.pdf]

Supplementary Information

A Skeletal Muscle Model of Infantile-onset Pompe Disease with Patient-specific iPS Cells

Takeshi Yoshida,[1,2] Tomonari Awaya,[2,3] Tatsuya Jonouchi,[1] Ryo Kimura,[3] Shigemi Kimura,[4] Takumi Era,[5] Toshio Heike,[2] Hidetoshi Sakurai[1,*]

[1]Center for iPS Cell Research and Application (CiRA), Kyoto University, Kyoto 606-8507, Japan

[2]Department of Pediatrics, Kyoto University Graduate School of Medicine, Kyoto 606-8507, Japan

[3]Department of Anatomy and Developmental Biology, Kyoto University Graduate School of Medicine, Kyoto 606-8501, Japan

[4]Kumamoto City Child Development Support Center, Kumamoto 862-0971, Japan

[5]Department of Cell Modulation, Institute of Molecular Embryology and Genetics (IMEG), Kumamoto University, Kumamoto 860-8556, Japan

[*]Correspondence: Hidetoshi Sakurai, M.D., Ph.D., Center for iPS Cell Research and Application (CiRA), Kyoto University, 53 Shogoin Kawahara-cho, Sakyo-ku, Kyoto 606-8507, Japan. Telephone: 81-75-751-3301; E-Mail: hsakurai@cira.kyoto-u.ac.jp

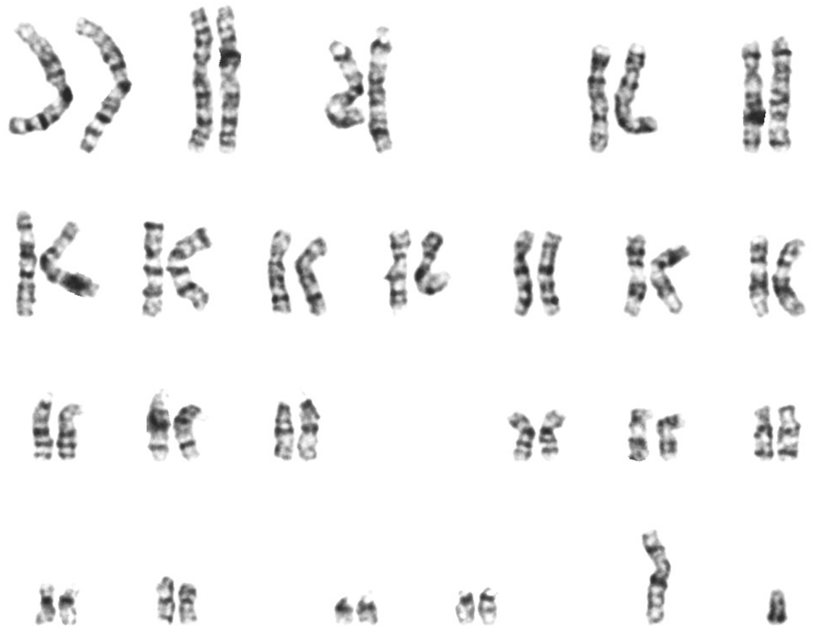

Supplementary Figure S1. Karyotype analysis by G-banding.

A representative image of G-banding in Pom1 iPSC line.

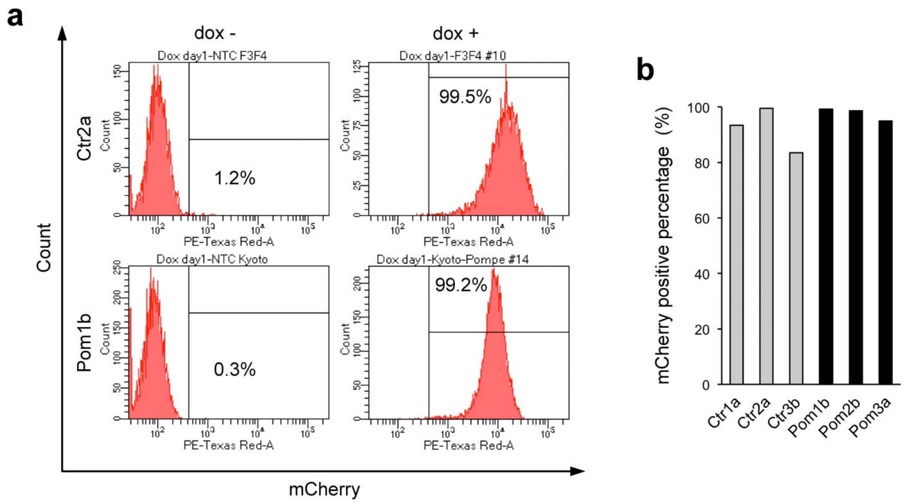

Supplementary Figure S2. Efficient transfection of *MyoD* into iPS Cells.

(**a**) Representative images of flow cytometric analysis for tetracycline-mediated mCherry expression at day 2 of myogenic differentiation. (**b**) Percentage of mCherry positive cells analyzed by flow cytometry at day 2 of myogenic differentiation with doxycycline.

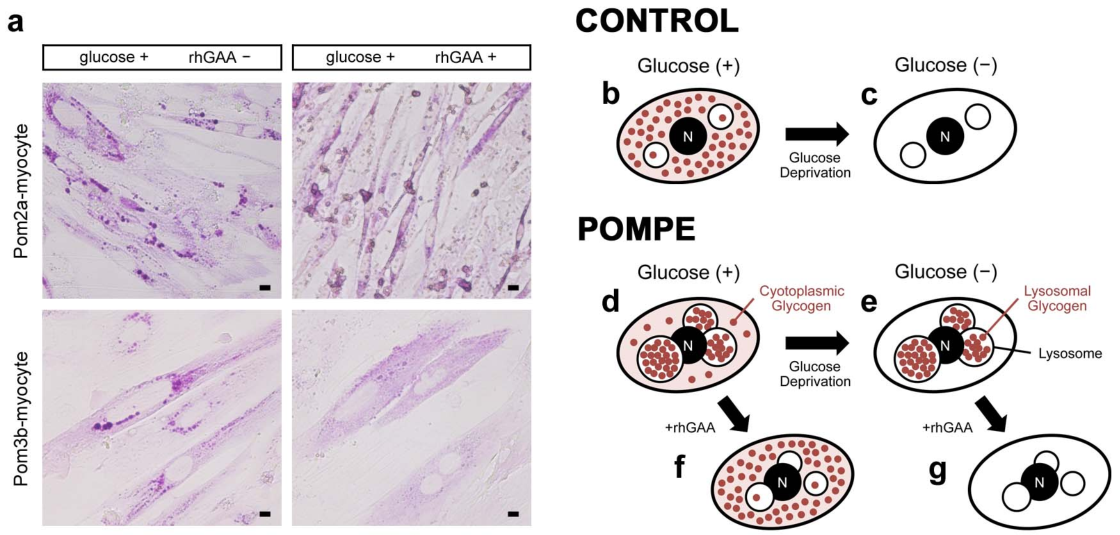

Supplemental Figure S3. Improvement of the lysosomal glycogen accumulation by rhGAA rescue in glucose-rich conditions.

(**a**) Bright field microscopic images of periodic acid-Schiff stain in representative Pom iPSC^{MyoD}-derived myocytes cultured in glucose-containing medium without rhGAA (left row) or treated for 3 days with rhGAA (right row). Scale bars = 10 μm. (**b-g**) Schematic overview of the glycogen state before and after glucose deprivation. Almost all the glycogen is detected in the cytoplasm in Ctr iPSC^{MyoD}-derived myocytes (**b**) and is consumed after glucose deprivation (**c**). In the case of Pom iPSC^{MyoD}-derived myocytes, accumulated lysosomal glycogen remains undegraded after glucose deprivation due to the lack of GAA (**e**). rhGAA degrades lysosomal glycogen into glucose; and the yielded glucose is released into the cytoplasm, where it is used to synthesize glycogen in glucose-rich conditions (**f**), or consumed in glucose-free conditions (**g**). A capital letter “N” represents a nucleus.

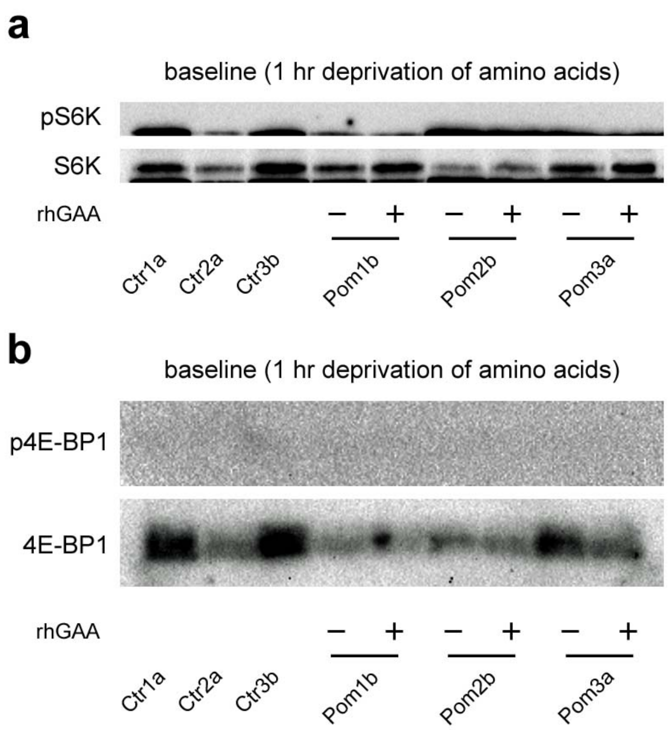

Supplemental Figure S4. S6K and 4E-BP1 are little phosphorylated before stimulation with amino acids and insulin.

Western blot analyses for pS6K and total S6K (**a**) and p4E-BP1 and total 4E-BP1 (**b**) after 1 hr deprivation of amino acids as a baseline in protein extracts from $iPSC^{MyoD}$-derived myocytes. Pom $iPSC^{MyoD}$-derived myocytes were treated with or without rhGAA.

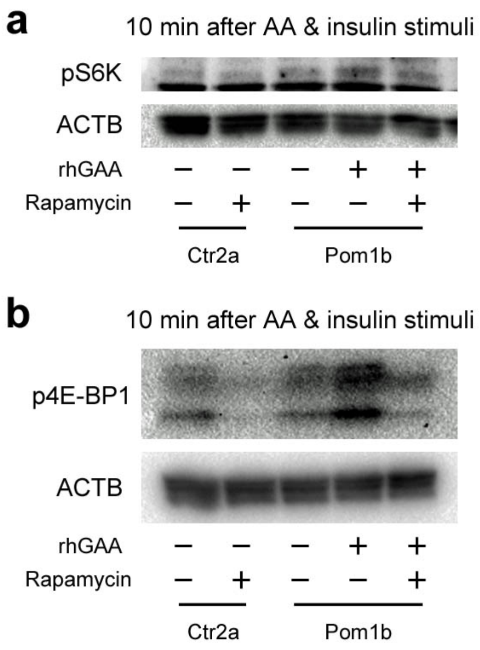

Supplemental Figure S5. Rapamycin pretreatment suppressed phosphorylation of S6K and 4E-BP1 by simulation with amino acids (AA) and insulin.

Western blot analyses for pS6K (A) and p4E-BP1 (B) after 10 min exposure to AA and insulin in protein extracts from iPSC^{MyoD}-derived myocytes. Myocytes were pretreated with 200 nM Rapamycin for 1 h ahead of stimulation. Pom iPSC^{MyoD}-derived myocytes were treated with or without rhGAA. Full-length membranes were presented below.

PCR Type	Name	FW/RV	Sequence (5’-3’)
RT-PCR	*Oct3/4*	FW	GACAGGGGGAGGGGAGGAGCTAGG
		RV	CTTCCCTCCAACCAGTTGCCCCAAAC
	Nanog	FW	CAGCCCCGATTCTTCCACCAGTCCC
		RV	CGGAAGATTCCCAGTCGGGTTCACC
	ACTB	FW	CTCTTCCAGCCTTCCTTCCT
		RV	CACCTTCACCGTTCCAGTTT
q-RT-PCR	*Myogenin*	FW	TGGGCGTGTAAGGTGTGTAA
		RV	CGATGTACTGGATGGCACTG
	MHC	FW	GCAGATTGAGCTGGAAAAGG
		RV	TCAGCTGCTCGATCTCTTCA
	CKM	FW	ACATGGCCAAGGTACTGACC
		RV	TGATGGGGTCAAAGAGTTCC
	UBQ-C	FW	GTAGTCCCTTCTCGGCGATT
		RV	TTGTCAAGTGACGATCACAGC

Supplemental Table S1. Primers for RT-PCR and q-RT-PCR analysis.

Abbreviations: q-RT-PCR, quantitative RT-PCR; FW, forward primer; RW, reverse primer; *ACTB, beta-actin*; *MHC*, *myosin heavy chain*; *CKM*, *creatine kinase M-type*; *UBQ-C, ubiquitin-c*.

Primary Antibody

	Dilution	Experiment	Company
SSEA4	1:100	IF	Merck Millipore, Billerica, MA, USA
TRA-1-60	1:100	IF	Merck Millipore, Billerica, MA, USA
TRA-1-81	1:100	IF	Merck Millipore, Billerica, MA, USA
LAMP2	1:100	IF	BD Biosciences, San Diego, CA, USA
MHC	1:200	IF	R&D Systems, Minneapolis, MN, USA
LC3	1:400	IF, WB	Sigma-Aldrich, St. Louis, MO, USA
pS6K	1:1000	WB	Cell Signaling Technology, Danvers, MA, USA
S6K	1:1000	WB	Cell Signaling Technology, Danvers, MA, USA
p4E-BP1	1:1000	WB	Cell Signaling Technology, Danvers, MA, USA
4E-BP1	1:1000	WB	Cell Signaling Technology, Danvers, MA, USA
HRP-ACTB	1:20000	WB	Sigma-Aldrich, St. Louis, MO, USA

Secondary Antibody

	Dilution	Experiment	Company
Alexa Fluor 488 goat anti-mouse IgG	1:500	IF	Thermo Fisher Scientific, Waltham, MA, USA
Alexa Fluor 488 goat anti-rabbit IgG	1:500	IF	Thermo Fisher Scientific, Waltham, MA, USA
HRP goat anti-rabbit IgG	1:10000	WB	Vector Laboratories, Burlingame, CA, USA

Supplemental Table S2. Primary and secondary antibodies used in this study.

Abbreviations: SSEA4, stage specific embryonic antigen 4; TRA, tumor-related antigen; LAMP2, lysosome-associated membrane protein 2; MHC, myosin heavy chain; LC3, microtubule-associated protein 1 light chain 3; S6K, p70 ribosomal S6 kinase; pS6K, phosphorylated S6K; 4E-BP1, eukaryotic initiation factor 4E-binding protein 1; p4E-BP1, phosphorylated 4E-BP1; HRP, horseradish peroxidase; ACTB, beta-actin; IF, immunofluorescence; WB, western blot.

Compound name	KEGG ID	HMDB ID	Ctr2a		Pom1b		Pom1b + rhGAA	
			Mean	S.D.	Mean	S.D.	Mean	S.D.
NAD^+	C00003	HMDB00902	1,457.12	293.67	1,317.16	137.19	2,088.31	30.47
cAMP	C00575	HMDB00058	4.93	1.59	7.69	2.42	10.29	0.76
cGMP	C00942	HMDB01314	N.D.		N.D.		N.D.	
NADH	C00004	HMDB01487	99.48	23.65	133.29	29.35	187.86	25.09
Xanthine	C00385	HMDB00292	N.D.	N.D.	N.D.	N.D.	N.D.	N.D.
ADP-ribose	C00301	HMDB01178	3.11	0.58	2.92	0.92	4.03	0.23
Mevalonic acid	C00418,C02104	HMDB00227	N.D.	N.D.	N.D.	N.D.	N.D.	N.D.
UDP-glucose	C00029	HMDB00286	1,775.77	370.69	1,092.08	33.40	1,805.70	124.43
Uric acid	C00366	HMDB00289	N.D.		N.D.		N.D.	
$NADP^+$	C00006	HMDB00217	26.95	3.95	26.38	1.47	39.59	3.99
IMP	C00130	HMDB00175	15.37	2.38	38.18	15.16	40.42	2.49
Sedoheptulose 7-phosphate	C05382	HMDB01068	N.D.		N.D.		N.D.	
Glucose 6-phosphate	C00668,C01172,C00092	HMDB01401	405.90	145.08	204.14	34.69	270.59	20.54
Fructose 6-phosphate	C05345,C00085	HMDB00124	80.67	28.06	38.07	3.18	46.42	1.20
Fructose 1-phosphate	C01094,C02976	HMDB01076	N.D.		100.56	100.07	50.01	15.87
Galactose 1-phosphate	C00446	HMDB00645	14.23	1.85	12.91	4.07	17.12	2.14
Glucose 1-phosphate	C00103	HMDB01586	40.94	13.84	48.79	14.83	66.78	6.66
Acetoacetyl CoA	C00332	HMDB01484	N.D.		N.D.		N.D.	
Acetyl CoA	C00024	HMDB01206	1.33	0.75	0.35	0.28	0.50	0.26
Folic acid	C00504	HMDB00121	7.96	2.42	5.99	3.10	8.46	0.89
Ribose 5-phosphate	C00117	HMDB01548	29.48	11.47	17.61	2.10	18.59	2.86
CoA	C00010	HMDB01423	20.35	7.97	18.49	3.17	35.95	9.23
Ribose 1-phosphate	C00620	HMDB01489	2.42	4.20	11.08	7.95	14.38	1.61
Ribulose 5-phosphate	C00199,C01101	HMDB00618	32.65	11.74	14.33	6.09	26.97	6.89
Xylulose 5-phosphate	C00231,C03291	HMDB00868	34.35	19.84	10.98	12.25	12.74	11.33
Erythrose 4-phosphate	C00279,C03604	HMDB01321	N.D.		N.D.		N.D.	
HMG CoA	C00356	HMDB01375	6.72	2.56	12.36	4.94	25.16	8.73
Glyceraldehyde 3-phosphate	C00118,C00661	HMDB01112	161.72	20.52	109.81	9.53	102.11	3.40
NADPH	C00005	HMDB00221	44.88	12.68	39.65	2.47	65.37	14.24
Malonyl CoA	C00083	HMDB01175	N.D.		N.D.		N.D.	
Phosphocreatine	C02305	HMDB01511	3,585.54	539.23	2,087.83	245.76	4,058.10	49.80
XMP	C00655	HMDB01554	4.59	2.20	5.23	0.94	7.17	1.06
Dihydroxyacetone phosphate (DHAP)	C00111	HMDB01473	554.01	63.29	492.22	93.58	560.78	60.16
Adenylosuccinic acid	C03794	HMDB00536	2.37	0.18	2.95	0.59	3.86	0.30
Fructose 1,6-diphosphate	C00354	HMDB01058	1,379.31	400.05	1,161.43	344.19	1,935.49	159.51
6-Phosphogluconic acid	C00345	HMDB01316	18.69	2.14	19.70	5.50	25.74	1.62
N-Carbamoylaspartic acid	C00438	HMDB00828	23.28	8.02	219.20	104.39	195.47	37.69
PRPP	C00119	HMDB00280	48.59	18.37	53.98	8.27	88.24	12.97
2-Phosphoglyceric acid	C00631	HMDB03391	9.58	2.20	13.35	2.51	21.83	0.34
2,3-Diphosphoglyceric acid	C01159	HMDB01294	63.96	24.65	70.54	18.85	116.62	8.50
3-Phosphoglyceric acid	C00197	HMDB00807	70.32	14.10	102.29	15.35	160.45	10.20
Phosphoenolpyruvic acid	C00074	HMDB00263	N.D.		N.D.		N.D.	
GMP	C00144	HMDB01397	41.99	13.11	88.09	8.19	114.34	3.45
AMP	C00020	HMDB00045	203.75	85.81	486.68	22.98	594.01	13.21
2-Oxoisovaleric acid	C00141	HMDB00019	N.D.		N.D.		N.D.	
GDP	C00035	HMDB01201	122.64	32.66	189.24	17.41	228.65	20.09
Lactic acid	C00186,C00256,C01432	HMDB00190,HMDB01311	11,315.48	1,626.74	17,006.39	2,466.63	22,933.36	1,120.08
ADP	C00008	HMDB01341	872.90	281.54	1,438.63	87.38	1,582.93	204.78
GTP	C00044	HMDB01273	1,506.78	307.18	1,392.41	206.62	2,237.59	165.85
Glyoxylate	C00048	HMDB00119	N.D.		N.D.		N.D.	
ATP	C00002	HMDB00538	10,253.92	1,997.54	8,814.10	690.50	14,797.53	1,192.35
Glycerol 3-phosphate	C00093	HMDB00126	2,431.30	214.13	3,499.16	2,241.69	3,116.69	200.17
Glycolic acid	C00160	HMDB00115	29.74	51.52	N.D.		N.D.	
Pyruvic acid	C00022	HMDB00243	83.65	74.52	38.02	65.85	118.20	33.02
N-Acetylglutamic acid	C00624	HMDB01138	51.67	3.34	122.04	45.42	155.45	9.82
2-Hydroxyglutaric acid	C02630,C01087,C03196	HMDB00606,HMDB00694	15.33	6.37	49.00	22.08	60.48	7.18
Carbamoylphosphate	C00169	HMDB01096	N.D.		N.D.		2.92	5.05
Succinic acid	C00042	HMDB00254	173.04	40.61	1,498.64	1,068.73	1,126.59	126.74
Malic acid	C00149,C00497,C00711	HMDB00156,HMDB00744	500.70	18.20	636.48	166.18	1,444.96	179.99
2-Oxoglutaric acid	C00026	HMDB00208	N.D.		N.D.		21.22	17.53
Fumaric acid	C00122	HMDB00134	76.94	0.96	94.99	6.62	227.43	60.55
Citric acid	C00158	HMDB00094	153.68	109.28	191.03	163.41	435.54	16.86
cis-Aconitic acid	C00417	HMDB00072	N.D.		N.D.		1.50	1.58
Isocitric acid	C00311	HMDB00193	N.D.		N.D.		N.D.	
Urea	C00086	HMDB00294	N.D.		N.D.		N.D.	
Glycine	C00037	HMDB00123	19,540.39	3,711.80	28,716.95	3,874.23	39,474.56	1,286.94
Putrescine	C00134	HMDB01414	87.44	4.87	239.90	79.43	317.88	40.34
β-Alanine	C00099	HMDB00056	488.69	177.31	367.42	61.22	529.43	19.77
Alanine	C00041,C00133,C01401	HMDB00161,HMDB01310	10,959.01	3,642.72	9,939.60	1,896.07	16,839.36	416.64
Sarcosine	C00213	HMDB00271	N.D.		N.D.		N.D.	
N,*N*-Dimethylglycine	C01026	HMDB00092	N.D.		N.D.		N.D.	
γ-Aminobutyric acid	C00334	HMDB00112	84.38	9.85	86.28	10.98	116.53	8.87
Choline	C00114	HMDB00097	643.68	188.20	392.17	399.39	235.49	33.73
Serine	C00065,C00716,C00740	HMDB00187,HMDB03406	13,986.61	749.00	14,559.97	1,893.87	19,705.15	1,471.59
Carnosine	C00386	HMDB00033	2.77	4.81	8.74	7.57	5.95	10.31
Creatinine	C00791	HMDB00562	14.13	9.25	9.12	0.36	18.28	2.92
Proline	C00148,C00763,C16435	HMDB00162,HMDB03411	26,693.64	2,513.27	44,157.28	6,043.59	59,177.43	3,972.01
Valine	C00183,C06417,C16436	HMDB00883	11,167.30	848.56	12,994.75	1,959.25	15,467.84	1,524.91

Betaine	C00719	HMDB00043	N.D.		N.D.		N.D.	
Threonine	C00188,C00820	HMDB00167	19,328.41	1,181.19	21,934.41	2,584.70	28,817.90	2,323.58
Homoserine	C00263	HMDB00719	N.D.		N.D.		N.D.	
Betaine aldehyde	C00576	HMDB01252	N.D.		N.D.		N.D.	
Cysteine	C00097,C00736,C00793	HMDB00574,HMDB03417	179.55	42.89	139.38	26.38	202.19	15.19
Hydroxyproline	C01015,C01157	HMDB06055,HMDB00725	684.87	27.43	776.61	127.65	997.84	164.94
Creatine	C00300	HMDB00064	11,982.78	2,108.85	11,445.92	2,313.93	16,550.11	960.09
Isoleucine	C00407,C06418,C16434	HMDB00172	12,437.52	1,162.30	14,416.51	1,765.52	17,356.16	1,546.98
Leucine	C00123,C01570,C16439	HMDB00687	4,622.43	585.57	5,533.56	986.31	6,722.52	731.80
Asparagine	C00152,C01905,C16438	HMDB00168	923.67	248.75	788.55	121.89	1,203.71	66.88
Ornithine	C00077,C00515,C01602	HMDB00214,HMDB03374	142.27	10.99	296.90	100.00	407.60	33.50
Aspartic acid (Asp)	C00049,C00402,C16433	HMDB00191,HMDB06483	12,446.19	3,606.89	5,897.66	1,311.39	9,543.54	282.74
Homocysteine	C00155,C05330	HMDB00742	N.D.		N.D.		N.D.	
Adenine	C00147	HMDB00034	N.D.		N.D.		N.D.	
Hypoxanthine	C00262	HMDB00157	N.D.		N.D.		N.D.	
Spermidine	C00315	HMDB01257	N.D.		N.D.		N.D.	
Glutamine	C00064,C00303,C00819	HMDB00641,HMDB03423	50,769.51	7,572.15	60,020.18	6,800.80	78,862.45	2,306.08
Lysine	C00047,C00739,C16440	HMDB00182,HMDB03405	2,296.70	415.13	3,388.17	862.41	4,127.13	518.42
Glutamic acid (Glu)	C00025,C00217,C00302	HMDB00148,HMDB03339	87,264.24	13,548.16	86,693.07	14,755.72	112,920.73	5,024.84
Methionine	C00073,C00855,C01733	HMDB00696	1,578.47	214.60	2,030.11	202.53	2,369.59	80.33
Guanine	C00242	HMDB00132	N.D.		N.D.		N.D.	
Histidine	C00135,C00768,C06419	HMDB00177	2,439.60	204.25	2,998.41	482.80	3,903.76	327.94
Carnitine	C00318,C00487,C15025	HMDB00062	N.D.		N.D.		N.D.	
Phenylalanine	C00079,C02057,C02265	HMDB00159	5,594.95	614.86	6,798.25	834.42	8,009.80	1,020.37
Arginine	C00062,C00792	HMDB00517,HMDB03416	963.68	186.05	1,390.36	323.52	1,668.22	302.12
Citrulline	C00327	HMDB00904	N.D.		N.D.		N.D.	
Tyrosine	C00082,C01536,C06420	HMDB00158	2,309.41	334.14	2,792.52	463.24	3,328.27	372.19
S-Adenosylhomocysteine	C00021	HMDB00939	N.D.		13.48	23.35	N.D.	
Spermine	C00750	HMDB01256	N.D.		N.D.		N.D.	
Tryptophan	C00078,C00525,C00806	HMDB00929	1,212.07	169.82	1,441.05	190.12	1,715.64	164.17
Cystathionine	C00542,C02291	HMDB00099	1,957.02	709.58	5,495.49	921.50	9,027.93	147.16
Adenosine	C00212	HMDB00050	N.D.		15.15	26.23	N.D.	
Inosine	C00294	HMDB00195	N.D.		N.D.		N.D.	
Guanosine	C00387	HMDB00133	N.D.		9.94	17.21	N.D.	
Argininosuccinic acid	C03406	HMDB00052	N.D.		N.D.		29.46	25.83
Glutathione (GSSG)	C00127	HMDB03337	6,555.05	1,628.70	5,002.19	1,081.33	6,282.12	736.80
Glutathione (GSH)	C00051	HMDB00125	12,908.79	1,880.63	11,375.36	3,294.00	17,615.37	755.14
S-Adenosylmethionine	C00019	HMDB01185	133.30	11.90	134.34	16.83	160.52	9.10
Adenylate Energy Charge	No ID	No ID	0.94	0.01	0.89	0.00	0.92	0.01
Total Adenylate	No ID	No ID	11,330.56	2,289.65	10,739.41	788.45	16,974.47	1,234.24
Guanylate Energy Charge	No ID	No ID	0.94	0.01	0.89	0.01	0.91	0.01
Total Guanylate	No ID	No ID	1,671.41	347.54	1,669.74	215.98	2,580.57	146.21
GSH/GSSG	No ID	No ID	2.01	0.26	2.45	1.30	2.84	0.42
Total Glutathione	No ID	No ID	26,018.89	5,125.03	21,379.74	1,794.13	30,179.60	909.05
NADPH/NADP+	No ID	No ID	1.65	0.30	1.51	0.14	1.65	0.26
NADH/NAD+	No ID	No ID	0.07	0.00	0.10	0.02	0.09	0.01
Glycerol 3-phosphate/DHAP	No ID	No ID	4.42	0.55	6.73	2.98	5.58	0.43
Total Amino Acids	No ID	No ID	286,713.34	41,196.39	326,630.76	37,708.28	431,415.95	21,713.65
Total Essential Amino Acids	No ID	No ID	60,677.44	5,304.98	71,535.23	8,381.54	88,490.34	8,157.31
Total Non-essential Amino Acids	No ID	No ID	226,035.89	35,967.44	255,095.53	29,326.84	342,925.61	13,570.74
Total Glucogenic Amino Acids	No ID	No ID	279,794.21	40,230.04	317,709.03	36,494.88	420,566.30	20,541.52
Total Ketogenic Amino Acids	No ID	No ID	47,801.48	4,405.49	56,304.47	6,312.20	70,077.42	6,592.51
Total Branched Chain Amino Acids	No ID	No ID	28,227.25	2,572.99	32,944.82	4,706.78	39,546.52	3,788.88
Total Aromatic Amino Acids	No ID	No ID	9,116.43	1,118.41	11,031.81	1,487.50	13,053.71	1,551.82
Fischer's Ratio	No ID	No ID	3.11	0.13	2.98	0.08	3.04	0.09
Total Glu-related Amino Acids	No ID	No ID	168,130.67	23,871.71	195,259.30	22,861.19	256,532.59	11,405.81
Total Pyruvate-related Amino Acids	No ID	No ID	65,206.04	9,446.71	76,731.37	9,873.75	106,754.79	4,777.52
Total Acetyl CoA-related Amino Acids	No ID	No ID	20,568.71	2,302.79	24,779.29	3,075.80	29,921.45	2,902.23
Total Fumarate-related Amino Acids	No ID	No ID	7,904.36	948.66	9,590.77	1,297.58	11,338.07	1,387.75
Total Succinyl CoA-related Amino Acids	No ID	No ID	25,183.29	2,177.54	29,441.38	3,921.32	35,193.58	3,147.02
Total Oxaloacetate-related Amino Acids	No ID	No ID	13,369.86	3,848.35	6,686.22	1,433.20	10,747.26	259.77
Malate/Asp	No ID	No ID	0.03	0.00	0.11	0.01	0.15	0.02
G6P/R5P	No ID	No ID	16.42	10.58	11.82	3.10	14.93	3.66

Supplemental Table S3. Metabolomic data in iPSC-derived-myocytes.

Abbreviations: S.D., standard deviation; N.D., not detected.

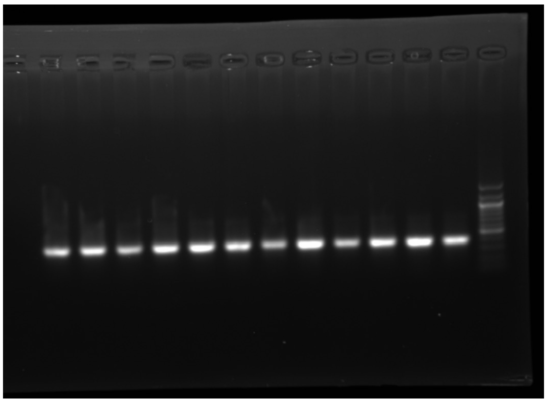

Nanog

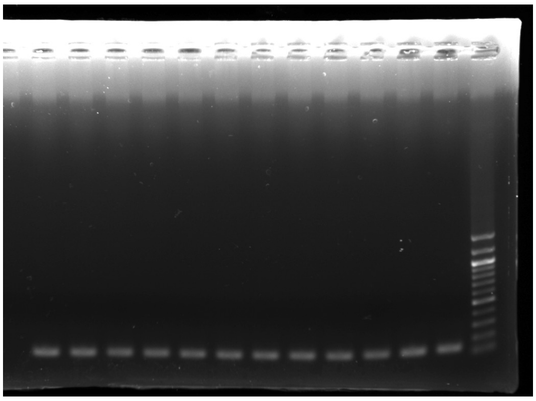

Oct3/4

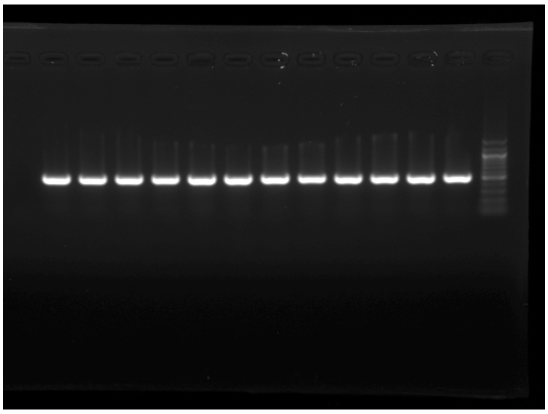

Beta-actin

Full-length gels of Figure 1b.

Lanes (from left to right): Ctr1a, b, Ctr2a, b, Ctr3a, b, Pom1a, b, Pom2a, b, Pom3a, b, Ladder.

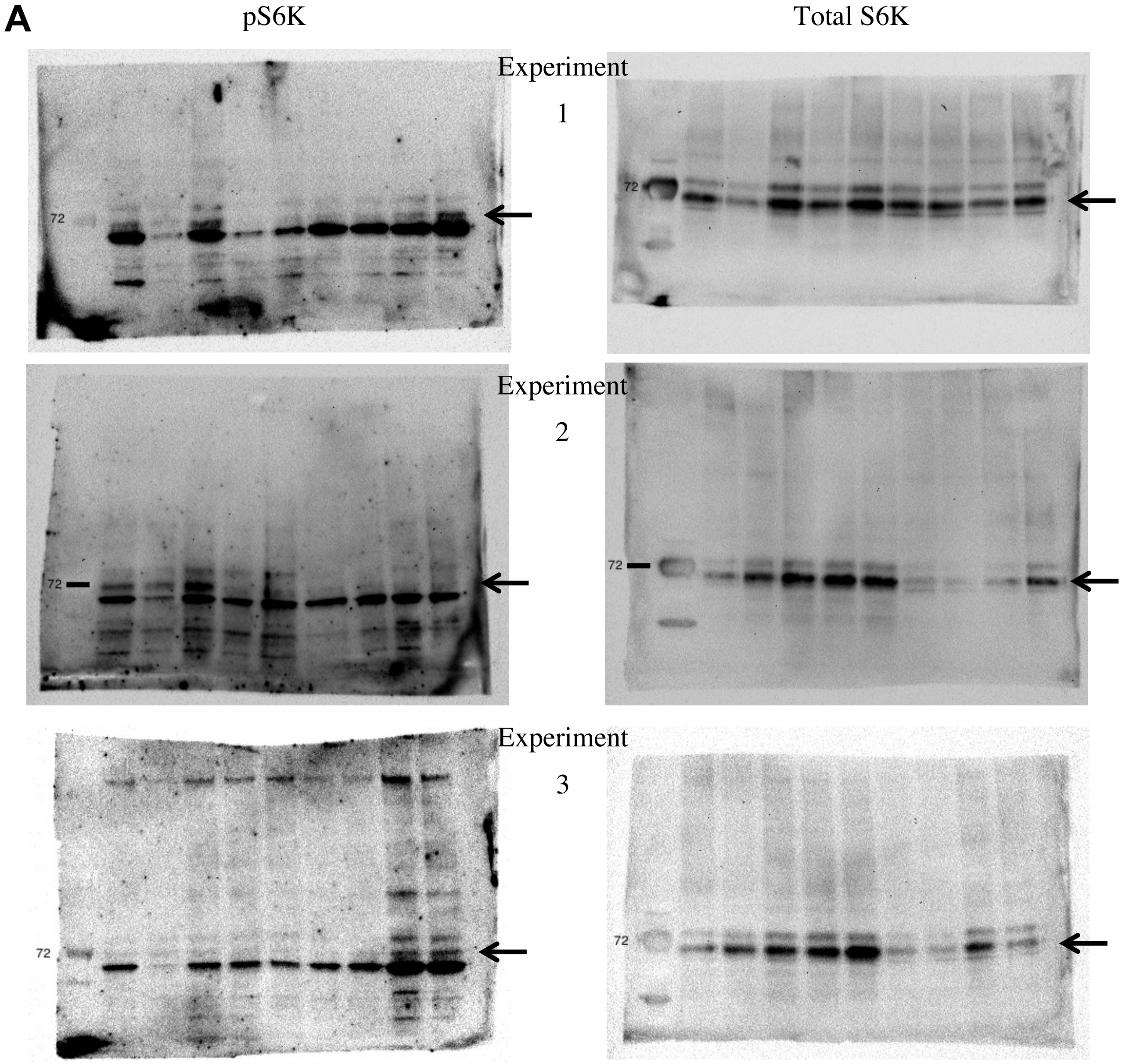

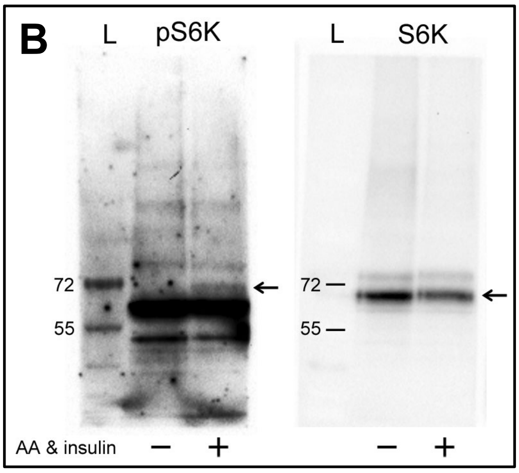

(**A**) **Full-length membranes of Figure 5a.** Left row, pS6K; Right, total S6K. Lanes (from left to right): Ladder, Ctr1a, Ctr2a, Ctr3b, Pom1b, Pom1b+rhGAA, Pom2b, Pom2b+rhGAA, Pom3a, Pom3a+rhGAA.

(**B**) **Confirmation of pS6K and S6K bands.** Western blot analysis for pS6K and total S6K without or with exposure to amino acid and insulin in Ctr2a iPSC^{MyoD}-derived myocytes. Abbreviations: L, ladder; AA, amino acid.

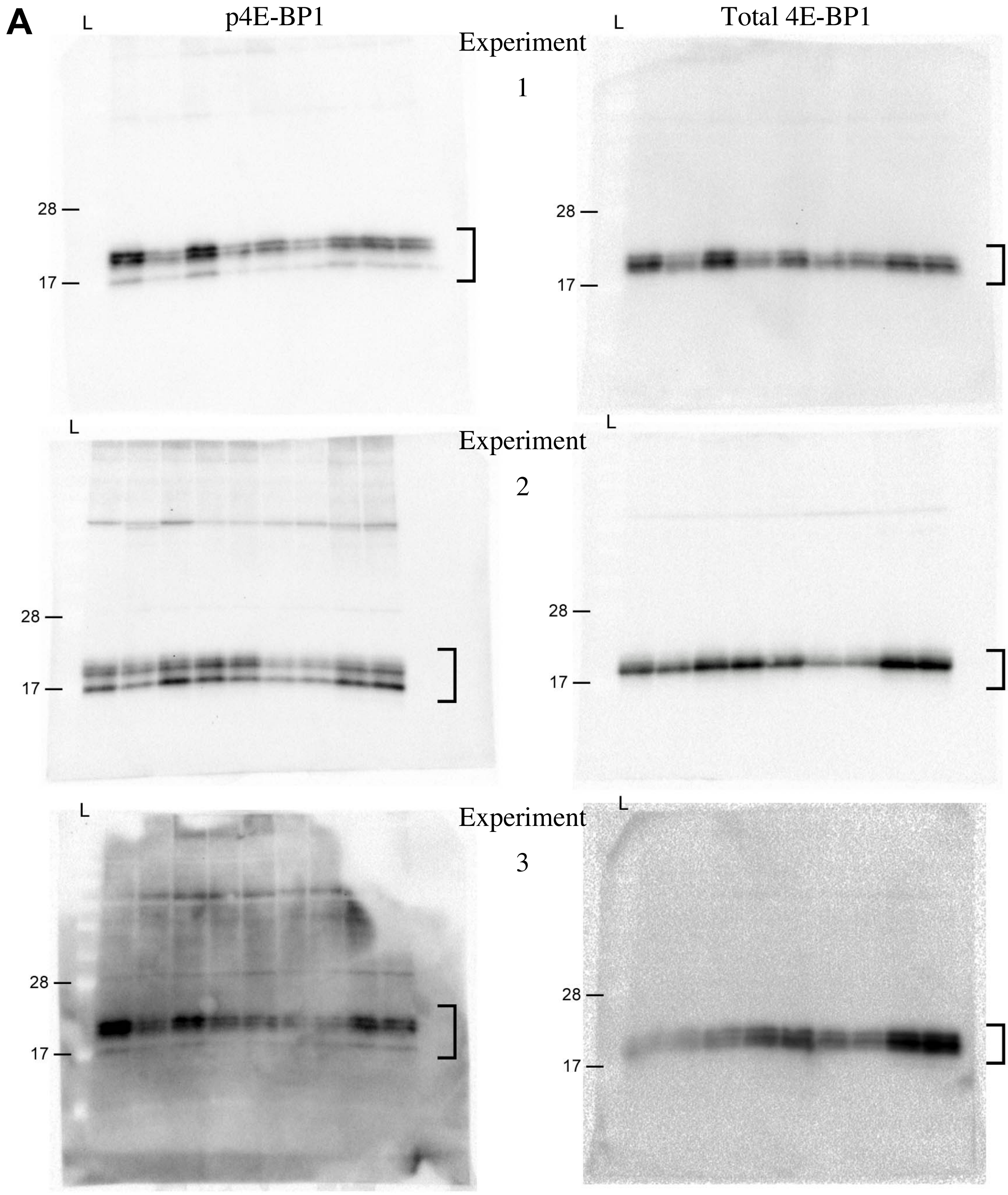

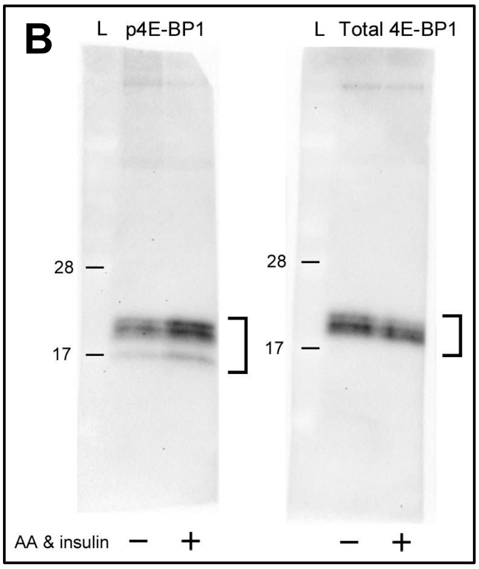

(**A**) **Full-length membranes of Figure 5c.** Left row, p4E-BP1; Right, total 4E-BP1. Lanes (from left to right): Ladder, Ctr1a, Ctr2a, Ctr3b, Pom1b, Pom1b+rhGAA, Pom2b, Pom2b+rhGAA, Pom3a, Pom3a+rhGAA.

(**B**) **Confirmation of p4E-BP1 and total 4E-BP1 bands.** Western blot analysis for p4E-BP1 and total 4E-BP1 without or with exposure to amino acid and insulin in Ctr2a $iPSC^{MyoD}$-derived myocytes.

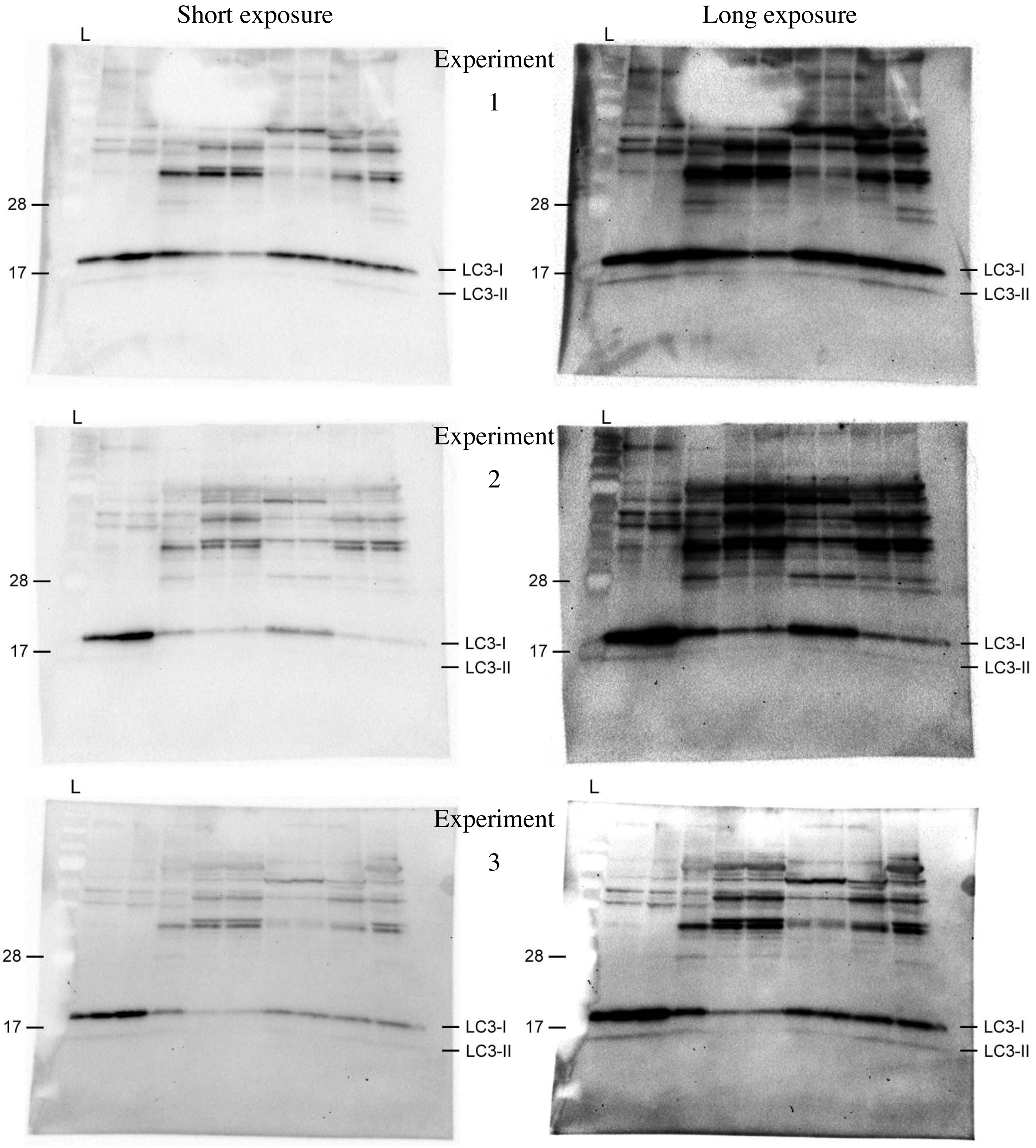

Full-length membranes of Figure 6c. Left row, short exposure; Right, long exposure. Lanes (from left to right): Ladder, Ctr1a, Ctr2a, Ctr3b, Pom1b, Pom1b+rhGAA, Pom2b, Pom2b+rhGAA, Pom3a, Pom3a+rhGAA.

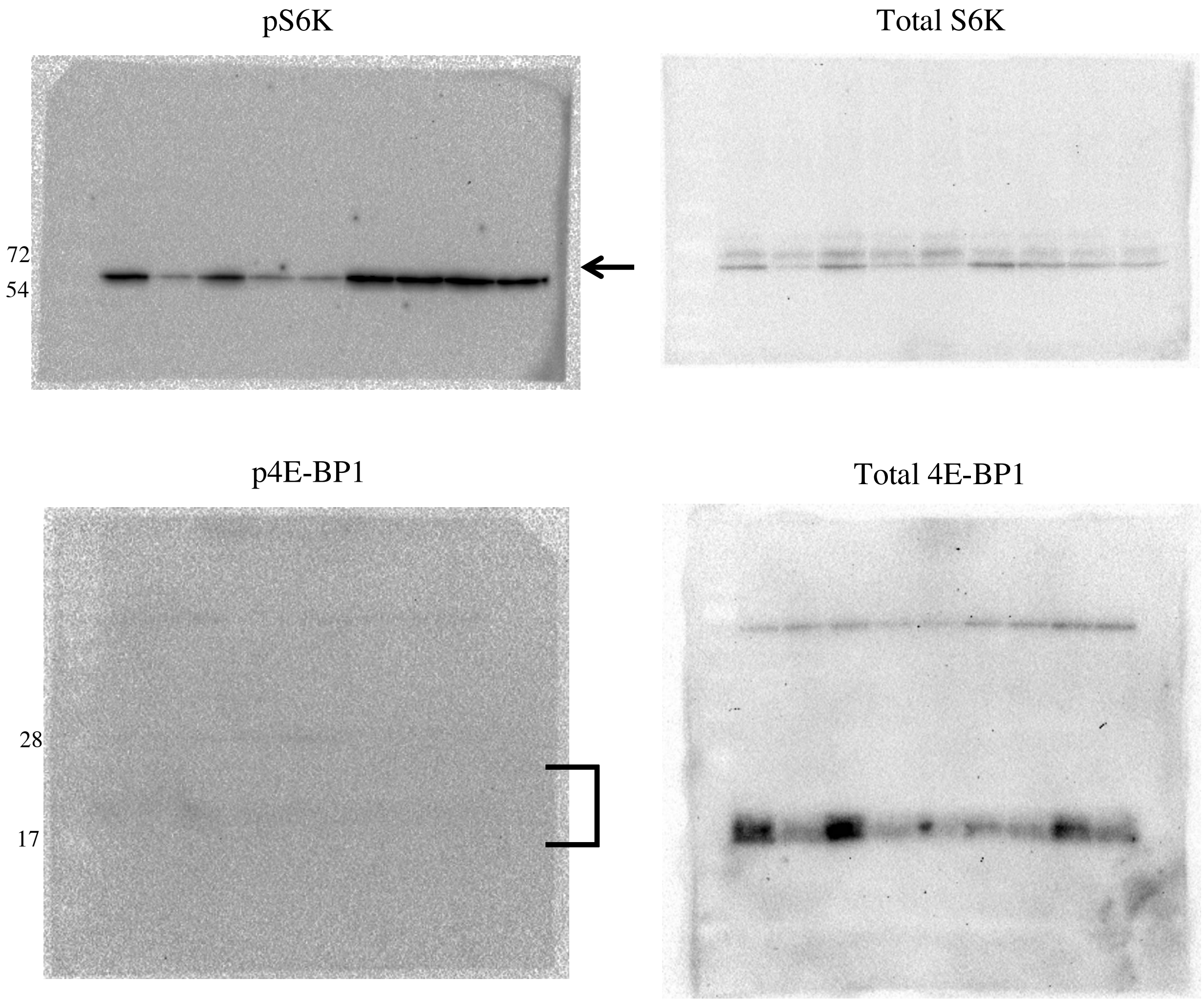

Full-length membranes of Supplementary Figure S4. Upper left panel, pS6K; Upper right, total S6K, Lower left, p4E-BP1; Lower right, total 4E-BP1. Lanes (from left to right): Ladder, Ctr1a, Ctr2a, Ctr3b, Pom1b, Pom1b+rhGAA, Pom2b, Pom2b+rhGAA, Pom3a, Pom3a+rhGAA.

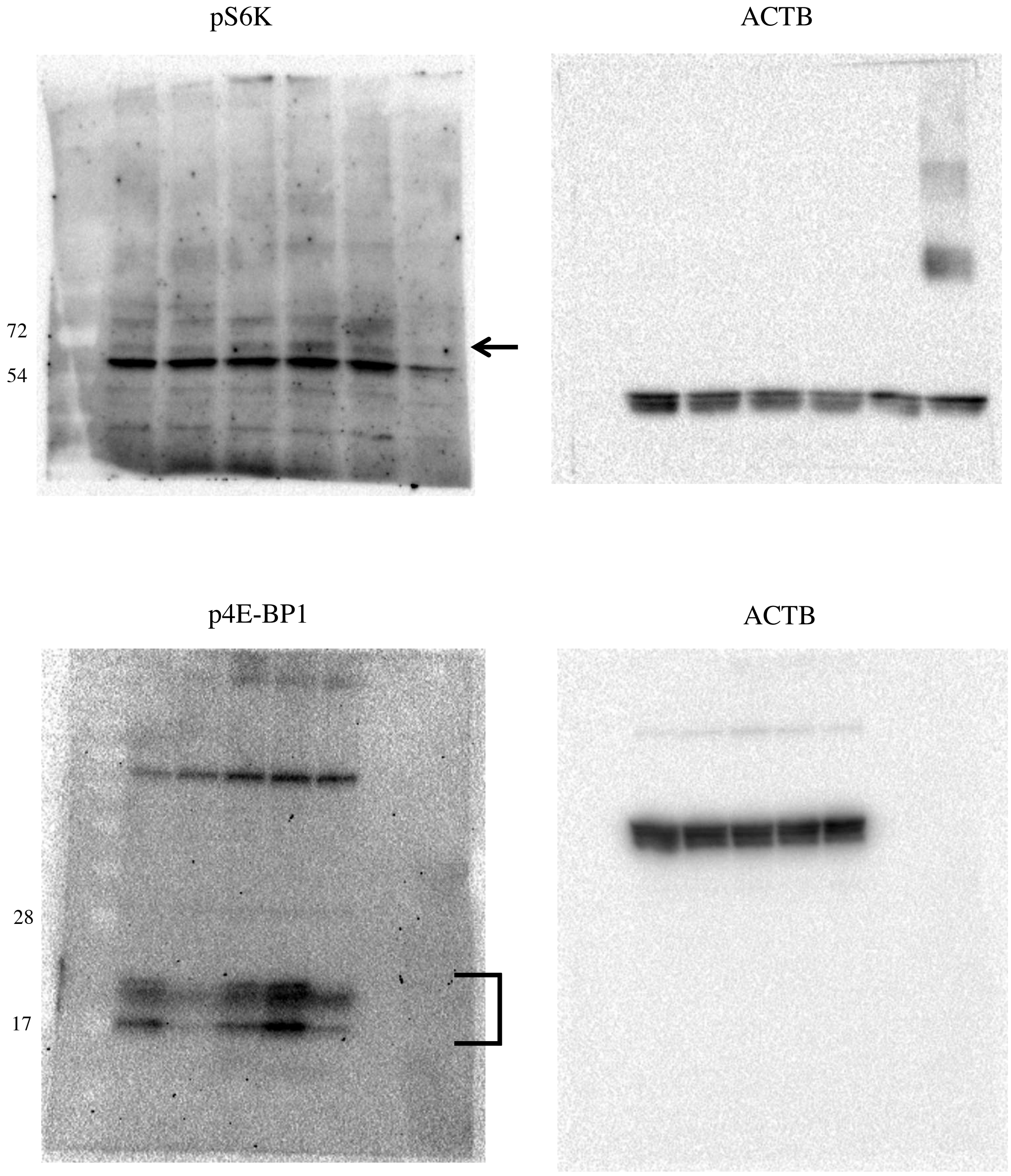

Full-length membranes of Supplementary Figure S5. Upper left panel, pS6K; Upper right, ACTB, Lower left, p4E-BP1; Lower right, ACTB. Lanes (from left to right): Ladder, Ctr2a, Ctr2a+Rapamycin, Pom1b, Pom1b+rhGAA, Pom1b+rhGAA+Rapamycin
